# Rationally designed carbohydrate-occluded epitopes elicit HIV-1 Env-specific antibodies

**DOI:** 10.1038/s41467-019-08876-w

**Published:** 2019-02-27

**Authors:** Cheng Zhu, Elena Dukhovlinova, Olivia Council, Lihua Ping, Edgar M. Faison, Shamit S. Prabhu, E. Lake Potter, Stephen L. Upton, Guowei Yin, James M. Fay, Laura P. Kincer, Ean Spielvogel, Sharon L. Campbell, S. Rahima Benhabbour, Hengming Ke, Ronald Swanstrom, Nikolay V. Dokholyan

**Affiliations:** 1 Department of Biochemistry and Biophysics, University of North Carolina at Chapel Hill, Chapel Hill, NC 27599 USA; 2 Departments of Pharmacology, Penn State College of Medicine, Hershey, PA 17033-0850 USA; 3 Lineberger Comprehensive Cancer Center, University of North Carolina at Chapel Hill, Chapel Hill, NC 27599 USA; 4 Department of Microbiology and Immunology, University of North Carolina at Chapel Hill, Chapel Hill, NC 27599 USA; 5 Division of Pharmacoengineering and Molecular Pharmaceutics, Eshelman School of Pharmacy, University of North Carolina at Chapel Hill, Chapel Hill, NC 27599 USA; 6 UNC-NCSU Joint Department of Biomedical Engineering, University of North Carolina at Chapel Hill, Chapel Hill, NC 27599 USA; 7 Departments of Biochemistry & Molecular Biology, Penn State College of Medicine, Hershey, PA 17033-0850 USA

## Abstract

An array of carbohydrates masks the HIV-1 surface protein Env, contributing to the evasion of humoral immunity. In most HIV-1 isolates ‘glycan holes’ occur due to natural sequence variation, potentially revealing the underlying protein surface to the immune system. Here we computationally design epitopes that mimic such surface features (carbohydrate-occluded neutralization epitopes or CONE) of Env through ‘epitope transplantation’, in which the target region is presented on a carrier protein scaffold with preserved structural properties. Scaffolds displaying the four CONEs are examined for structure and immunogenicity. Crystal structures of two designed proteins reflect the computational models and accurately mimic the native conformations of CONEs. The sera from rabbits immunized with several CONE immunogens display Env binding activity. Our method determines essential structural elements for targets of protective antibodies. The ability to design immunogens with high mimicry to viral proteins also makes possible the exploration of new templates for vaccine development.

## Abstract (web summary)

Areas of HIV envelope (Env) that aren’t covered by glycans are potential targets for antibodies. Here, the authors computationally design small protein mimics of four such epitopes and show that they can induce Env binding antibodies in rabbits.

## Introduction

HIV-1 carries on a continuous battle with the host immune system^1,2^. As the sole target of neutralizing antibodies, the virion surface protein Env encodes a glycan shield to restrict the antibody access to antigenically conserved sites^3,4^. There are about 30 sites of carbohydrate addition on each HIV-1 Env protomer, and about two-thirds of the N-linked carbohydrates cover the generally conserved outer domain of Env^5,6^. This glycan shield serves as a barrier to an antibody response that would otherwise be directed at surface features of Env^7–9^. Variation in carbohydrate addition sites has been documented wherein 90% of HIV-1 strains are missing at least one conserved glycosylation site^10,11^. When Env trimers from different HIV-1 clades (A, B, and C) were used as immunogens, the autologous neutralizing antibody response was targeted to the protein face at the site of missing glycans^12,13^. Similarly, the SIV variants missing a dispensable glycan were used to infect macaques and gave rise to an antibody response that targets the exposed area^3,14^. Infection with a virus missing a glycan on the Env α2 helix led to the development of an antibody escape mutant that reacquired the original glycosylation site, suggesting that antibodies to such surface features of Env can provide selective pressure and thus be protective^15^. It is these types of carbohydrate-occluded structural features we refer to as CONEs. We reason that HIV-1 isolates present a collective vulnerability at the surface features under variable glycosylated sites. We set out to exploit their immunogenic nature by eliciting antibodies that interact specifically with individual CONEs.

**Fig. 1:**
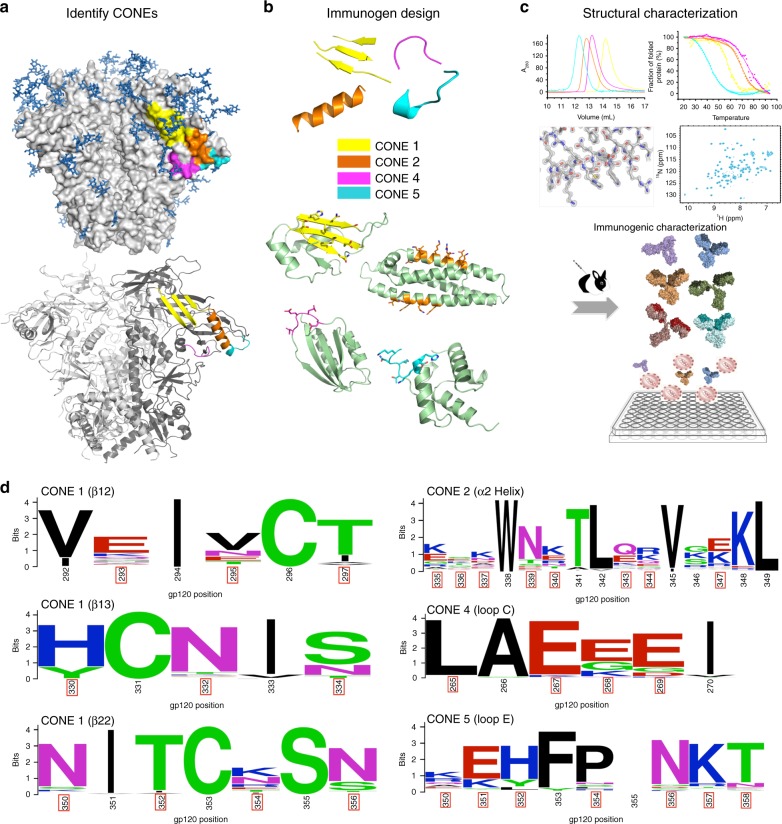
Rational design of HIV-1 immunogens and elicitation of antibodies targeting the CONEs. **a** Fully glycosylated Env (upper panel) encodes a shield of glycan (dark blue) masking its conserved surface. In underglycosylated Env (lower panel) the carbohydrate-occluded neutralization epitopes (CONEs) are exposed. The transmitted HIV-1 viruses isolated from infected individuals are typically missing at least one carbohydrate in one CONE (shown in colors, see key in panel **b**). **b** Small protein mimics of the CONEs, designed by epitope transplantation, can serve as potential immunogens to focus the antibody response towards CONEs. **c** The experimental workflow includes biophysical characterization (circular dichroism, size exclusion chromatography), structural characterization (X-ray crystallography, NMR) of designed proteins, and immunogenic evaluation in animal models. **d** Consensus protein sequences of the CONEs. The site convergence data were derived from 183 Env sequences of clade C HIV-1 isolated from acutely infected patients. The solvent-exposed resides in Env are marked with red boxes

We previously examined the *env* gene of HIV-1 subtype C, which accounts for ~50% of new infections worldwide, including samples from acutely and chronically infected individuals^10^. Our results demonstrated moderate conservation of twenty-two N-linked glycosylation sites on the Env outer face, including positions 130, 230, 234, 289, 332, 337, 356, and 442 (HXB2 numbering), with each glycosylation site appearing in 65–85% of HIV-1 isolates. We find that seventeen of these moderately conserved glycosylation sites cluster around six surface structural features, and we hypothesize the absence of a surface glycan at any one of these CONEs would expose the underlying protein structural elements (Fig. 1a and Supplementary Fig. 1). Analysis of transmitted HIV-1 isolates revealed that 93% were missing at least one carbohydrate in one of the CONEs, with 80% missing carbohydrates in two or more CONEs^10^. In previous studies, others have built structural mimetic of CONE 3 (a four-stranded β sheet at the base of the V1/V2 loops) and CONE 6 (the CD4 binding site) for structure-guided immunization. Chimeric glycoproteins encoding CONE 3 bind the broadly neutralizing antibody PG9^16^. Protein nanoparticles containing CONE 6 mimetics engage the germline precursors of VRC01-class neutralizing antibodies and direct the evolution of antibodies against the CD4 binding site^17,18^. Here we focus our protein design efforts on four CONEs, including the structural elements of β sheet 12/13/22 (CONE 1), α2 helix (CONE 2), loop C (CONE 4), and loop E (CONE 5) (Fig. 1b). Small protein mimics of the CONEs are designed by epitope transplantation and used as immunogens to focus the antibody response^19–21^. Our experimental workflow includes biophysical and structural characterization of the designed proteins, followed by immunogenic evaluation in animal models (Fig. 1c).

## Results

### Structure-based immunogen design

Atomic-level details of the fully glycosylated, pre-fusion conformation of the Env trimer have been obtained as crystal and cryo-electron microscopy structures^5,22^. The conformations of the CONEs are well conserved in the structures of Env from clades A, B, C, and G (Supplementary Fig. 1b). We identified the substructural elements of each CONE based on the BG505 SOSIP.664 trimer structure (Protein Data Bank [PDB]: 4TVP and 4NCO) and adopted the consensus sequence of clade C HIV-1 Env as the reference for the immunogen design^23,24^ (Fig. 1d and Supplementary Fig. 2). The rationales for identifying CONEs include: (i) the change of solvent-exposed area on the Env surface due to absence of glycans. The boundaries of CONE residues can be determined by modeling the interactions between underglycosylated Env and immunoglobulin (Supplementary Fig. 3). (ii) whether the sub-structures of CONEs can be readily matched to existing scaffolds in PDB (see Methods). (iii) conservation of surface glycan^10,11^. Using a rapid structural motif-mining algorithm *Erebus*^25^ (Supplementary Fig. 4, workflow), we searched the PDB for scaffolds capable of supporting the disembodied CONE structures (three-stranded β-sheet, α-helix, and two separate loops). We estimated the surface matching between scaffolds and CONEs by analysis of root-mean-square deviations (RMSD, 0.5–3.0 Å) and selected the top-ranking scaffolds for each CONE (Supplementary Table 1). The surface amino acids of the CONE-like region within each scaffold were replaced by solvent-exposed residues in the consensus sequences of CONEs (Supplementary Figs 2 and 4). For instance, a discontinuous three β-strand motif was grafted onto CONE 1 scaffolds, while the two helixes on the CONE 2 and scaffold 5 design (C2S5, a similar nomenclature is followed for all designed proteins) displayed the α2 helix-specific residues (Supplementary Fig. 5). The modified scaffold was subsequently relaxed by discrete molecular dynamics^26^.

For each scaffold, multiple designs (86 in total) were generated adopting various combinations of CONE residues and their substitutes in the consensus sequences. We then estimated the change in free energy of these substitutions (ΔΔ*G*_mut_) using the *Eris* molecular design suite and discarded highly destabilizing mutations (55 designs of ΔΔ*G*_mut_ > 6 kcal mol^−1^)^27^. The conformational stabilities of all CONE mimetics were further evaluated through molecular dynamics simulations, which allowed us to rapidly screen the designed protein library and choose the scaffolds that exhibited substantial rigidity around the CONE segment (root-mean-square fluctuations (RMSF) analysis, Supplementary Fig. 6). A recent study of massively parallel protein design demonstrated that structural elements of the most successful designs exhibit high similarity to natural proteins of similar local sequences^28^. Hence, we reasoned that the success of epitope transplantation would correlate with the compatibility between local structure of grafted sequences and their native conformation in Env, whereas higher RMSF of grafted residues indicates that the design explores wider conformational states and likely displays poor agreement to the target structure. Therefore, we filtered out the designs featuring significant plasticity of the grafted epitopes or designated them as alternative scaffolds (21 designs of RMSF > 3.5 Å at CONEs). Overall, three to five designs of each CONE were expressed in *Escherichia coli* (Supplementary Fig. 5). Of the sixteen candidates tested (one most stable design for each scaffold), eight could be successfully purified as soluble proteins (Supplementary Table 2).

### Biophysical characterization of designed proteins

The eight proteins exhibited circular dichroism (CD) spectra consistent with the designed topology (Supplementary Fig. 7a). The stabilities of the designs to thermal denaturation were assessed by CD spectroscopy. Their melting temperatures (*T*_m_) were in the range of 42–75 °C (Supplementary Fig. 7b). The CONE 1, 2, and 4 proteins were monomeric in solution (Supplementary Fig. 7c, d), while the C5S3 design displayed rapid exchange between monomeric and dimeric states. We selected the most stable designs (C1S1, C2S5, and C4S3) for immunization experiments and further structural analysis.

**Fig. 2:**
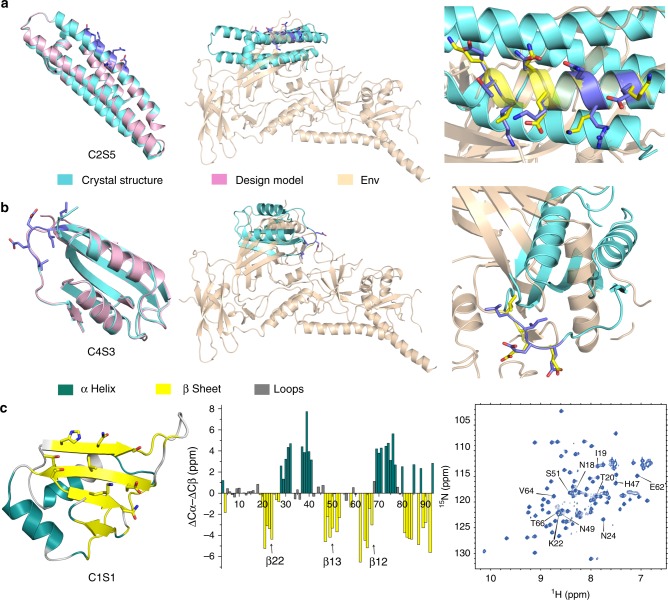
Structural characterization of designed CONE immunogens. **a** Crystal structure of C2S5 (PDB 6CFE) aligned to the design model (left) and to the gp120 CONE 2 region (middle, PDB 5FYL, clade A sequence), with the epitope shown as sticks; (Right) Close-up view of the alignment between grafted resides in C2S5 (slate) and the gp120 (yellow) residues in their native conformations. **b** Crystal structure of C4S3 (PDB 6CBU) with the CONE 4 epitope shown as sticks (slate). **c** Secondary structure assignment based on solution NMR mapped to the C1S1 design model (left). Plot of the differences of chemical shifts ΔCα−ΔCβ (middle) for each residue of C1S1 indicates which residues adopt α-helical (>1 ppm, dark cyan) or β-sheet (<−1 ppm, yellow) structures. (Right) ^1^H-^15^N HSQC spectrum with the assignments to CONE 1 residues indicated. Source data are provided as a Source Data file. See also Supplementary Fig. 9

To determine whether the designs recapitulate the native conformations of each CONE, we solved the crystal structures of C2S5 (α2 helix) and C4S3 (loop C) at resolutions of 2.0 Å and 1.2 Å, respectively (PDB 6CFE and 6CBU, Table 1). The electron density maps revealed that the side chains of the grafted residues were well positioned relative to the computational model (Supplementary Fig. 8). Comparison with the Env trimer protein revealed a high degree of mimicry: within the epitope region, the Cα RMSDs between the designed proteins and Env were 0.42 Å (C2S5) and 0.34 Å (C4S3), respectively, suggesting that our CONE mimetics accurately display the Env residues (Fig. 2a, b). The C2S5 scaffold is built on a four-helix bundle from apolipoprotein E3. The two helixes used to display the CONE 2 residues were well matched to the α2 helix in terms of the Cα-Cβ orientations (Fig. 2a and Supplementary Fig. 8d). The CONE 4 (loop C) residues were grafted onto the C-terminal loop region of an α/β mixed protein (Fig. 2b). A characteristic array of three glutamic acids flanked by hydrophobic residues defines the conformation of CONE 4 loop in Env. Our design largely recapitulates the conformations of two charged side chains (E88 and E89). The hydrophobic residue L86 also adopts a similar conformation to L265 of Env (Fig. 2b). Consistent with the molecular dynamics simulation, we found that the C4S3 loop adopted a relatively rigid structure, as assessed by the low B-factors (8–12 Å^2^, Supplementary Fig. 6).

**Table 1:** Crystallographic data collection and refinement statistics

	C2S5	C4S3
*Data collection*		
Space group	P3_1_21	P2_1_2_1_2_1_
Cell dimensions		
* a*, *b*, *c* (Å)	46.88, 46.88, 140.42	26.07, 57.61, 62.94
* α*, *β*, *γ* (˚)	90, 90, 120	90, 90, 90
Resolution (Å)	50.00–2.00 (2.03–2.00)^a^	50.00–1.20 (1.22–1.20)
*R*_merge_	0.064 (0.886)	0.056 (0.130)
*I* / *σI*	13.6 (3.7)	18.9 (6.9)
Completeness (%)	99.67	98.40
Redundancy	18.0 (16.5)	14.1 (13.9)
No. of unique reflections	12904	30098
*Refinement statistics*		
Resolution (Å)	39.00–2.00	42.49–1.20
No. of reflections	12849	28610
*R*_work_/*R*_free_ (%)	23.65/26.87	14.30/15.30
No. of atoms		
Protein atoms	2344	771
Ligand/ion	0	1 (SO_4_^2−^)
Water	17	67
Average *B*-factor (Å^2^)		
Protein	70.43	7.54
Ligand/ion	NA	7.69
Water	48.78	14.10
RMS deviation from ideality		
Bond lengths (Å)	0.004	0.006
Bond angles (°)	0.513	1.198
Ramachandran statistics^b^		
Favored regions (%)	99.28	89.20
Allowed regions (%)	0.72	10.80
Outliers (%)	0	0

^a^Highest resolution shell statistics are shown in parentheses

^b^As defined by MolProbity

We also acquired structural information for C1S1 using nuclear magnetic resonance (NMR). The two-dimensional ^1^H-^15^N heteronuclear single-quantum coherence (HSQC) spectrum suggests that C1S1 is well folded in solution (Fig. 2c, right panel). We were able to assign 72 of 77 peaks corresponding to C1S1 residues with the aid of the HNCACB and CBCA(CO)NH spectra (Supplementary Fig. 9). The backbone information derived from Cα/Cβ chemical shift indicates that C1S1 possesses four β-strands (three from CONE 1 and one from the scaffold protein: hnRNP RNA-binding domain) and two α-helices, as expected based on the design model (Fig. 2c, left panel). All CONE 1-associated residues adopted a β-strand conformation (middle panel).

### Immunogenic evaluation of CONE immunogens

**Fig. 3:**
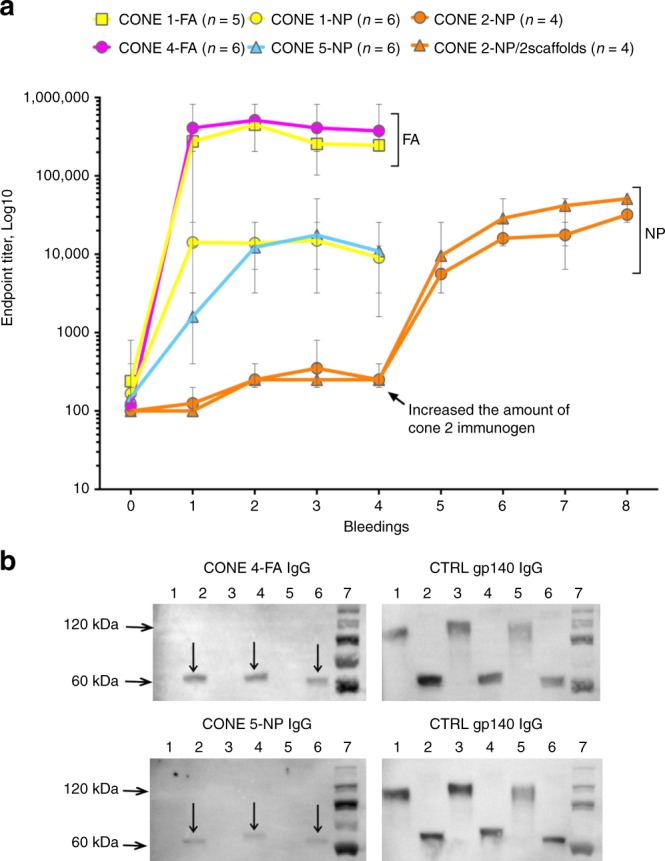
Characterization of antibodies in the sera from rabbits immunized with CONEs. **a** Anti-CONE endpoint titers were determined by ELISA during the immunization process. The data represent the mean titers for each group of rabbits. The boost injections were performed monthly, with bleedings performed three weeks after each boost. *n* = number of rabbits (4–6, indicated for each immunogen). Error bars represent standard deviation. **b** Western blots demonstrating the binding of purified rabbit IgG to PNGase-deglycosylated gp120 (~60 kDa band, lanes 2, 4, and 6 in plots of CONE 4 and 5). No interaction between the IgG and glycosylated gp120 (~120 kDa band, lanes 1, 3, and 5 in plots of CONE 4 and 5) was observed. (Left) blots treated with IgG purified from rabbits immunized with CONEs; (Right) same blots stripped from CONE IgG and treated with anti-gp140 IgG as a positive control. Lanes: 1–2, 1086 gp120 D7 293F; lanes 3–4, C.TV1C8.2D11 gp120; lanes 5–6, CHO monomer 1086 D7 gp120 K160N; lane 7, PageRuler Plus Prestained Protein Ladder. Source data are provided as a Source Data file

We next immunized small groups of rabbits (four to six per group) with the designed immunogens to raise CONE-specific antibodies. Two different immunization strategies were applied: incomplete Freund’s adjuvant (FA) and a nanoparticle (NP) formulation without adjuvant (Supplementary Tables 3, 4, Supplementary Fig. 10). In these initial immunization experiments, we sought a qualitative understanding of the nature of the antibodies that can be generated to each CONE. The CONE 1, 2, 4, and 5 mimetics were all immunogenic, giving rise to autologous binding antibodies as determined by enzyme-linked immunosorbent assay (ELISA, Fig. 3a). The FA-associated immunization protocols induced antibodies with the titers more than 10-fold higher than those induced by NP-associated immunization. As a control experiment, we also immunized five rabbits with a synthetic peptide of the CONE 4 sequence (LAEEEI). None of these rabbits could induce antisera of significant titers (Supplementary Table 5), in contrast to the C4S3-immunized rabbits, indicating that for a linear epitope, constraining it in the right conformation with suitable protein scaffolds is favorable for the generation of functional antibodies.

We then evaluated antibody specificity for the CONE-derived epitopes (Supplementary Fig. 11). For each immunogen, an alternative design (C1S5, C2S3, C4S2, and C5S2, Supplementary Table 2) with the same CONE grafted onto a different scaffold protein was used as a control to HIV-specific responses. No HIV-specific antibodies were obtained after immunization with CONE 2 (α2 helix). The CONE 4 (loop C) and CONE 5 (loop E) immunogens did induce HIV-specific antibodies as assessed by ELISA and western blot assay. The purified IgG fractions of anti-CONE 4 and anti-CONE 5 rabbit sera bound to the deglycosylated form of gp120 but not the glycosylated protein (Fig. 3b).

We also analyzed the anti-Env responses of individual rabbit sera in an ELISA assay using the deglycosylated Env SOSIP trimer. We modified the sequence of SOSIP from the original BG505 SOSIP.664 isolate^23^, so that the exposed residues at the CONE regions represent the consensus sequences of Env subtype C. The sera of the rabbits immunized with C1S1 (FA protocol) contained antibodies that bound Env, featuring a significantly elevated response to Env in comparison to the homologous pre-immune sera; in contrast, only one animal generated strong-binding antibodies with C1S1-NPs (Supplementary Fig. 12). Most of the animals immunized with C4S3 also generated antibodies that bound to the deglycosylated SOSIP Env protein, whereas no such antibodies were detected in animals immunized with C5S3.

**Fig. 4:**
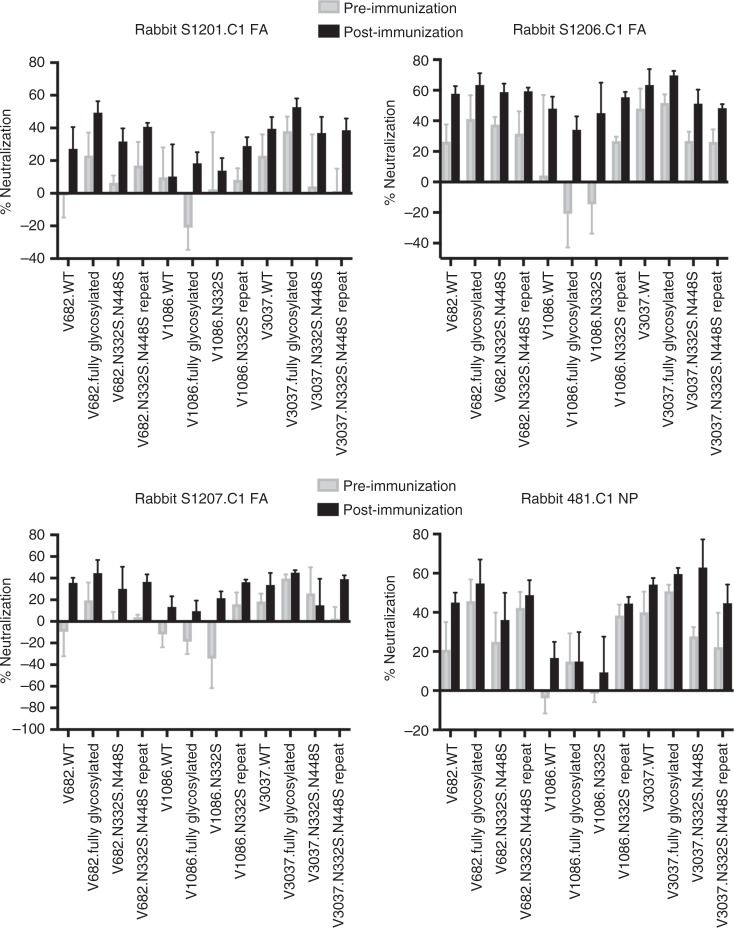
Anti-CONE 1 rabbit sera have moderate inhibitory activity against pseudotyped HIV-1. Pre- and post-immunization sera were heat inactivated, diluted 1:10 in media, and incubated with pseudotyped HIV-1 for 2 h before addition to TZM-bl cell culture. The inducible luciferase activity was quantified as a surrogate of viral infectivity. Percent neutralization (%) was determined by subtracting the pre- and post-immunization relative light units (RLU) from the virus-only RLU (not shown) and dividing the differences by the virus-only RLU. Each experiment was repeated three times. Pseudotyped HIV-1 isolates included in the assays: V682, V1086, and V3037. Rabbits ID: S1201, S1206, S1207 and 481. *n* = 3 independent experiments. Error bars represent standard deviation. Source data are provided as a Source Data file

Four C1S1-immunized rabbits generated sera with the highest reactivity to the SOSIP trimer. We tested these serum samples in a virus neutralization assay against reporter HIV-1 viruses, which were pseudotyped using three different *env* gene clones (682, 1086, and 3037, all subtype C). The viruses expressing the WT Env represent the samples isolated from infected subjects, while the fully glycosylated Env and the mutants represent the CONE-concealed and CONE-revealed samples, respectively (Fig. 4). In this analysis, we accounted for the animal-to-animal variation by comparing responses of the pre-immune serum and the immunization endpoint serum for each rabbit and each virus. The four post-immunization rabbit sera exhibited modest virus inhibitory activity in comparison to the homologous pre-immune serum. The highest responses we observed (50–70% inhibition) were with virus 3037 (missing N332 and N448 glycans) by the serum of rabbit 481, and with virus 1086 (missing N332 glycan) by the serum of rabbit S1206. Given the low level of virus inhibition associated with these sera it is difficult to distinguish if this activity represents true virus neutralization or some type of nonspecific inhibition of infectivity, although we note this activity is differentially present after immunization. The observed inhibitory activity did not depend critically on the absence of the CONE 1-associated glycans on the Env protein surface. If this does represent antibody-mediated neutralization, then the rabbit-raised CONE 1-specific antibodies may possess long loops in the complementarity-determining region that are capable of penetrating the glycan shield, as seen in various neutralizing antibodies characterized previously^1,7,8^. In this scenario the occluded surface features may not be immunogenic under the glycan shield, but the corresponding antibodies when they exist may be able to intercalate between structurally dynamic glycan side chains, thus resulting in neutralization.

## Discussion

The strategies that HIV-1 adopts to avoid in vivo neutralization, including hypervariable protein sequences and a glycan coat that occludes the conserved protein surface, confounded the attempts at immunization using Env trimer as antigens^29,30^. Here we described the implementation of an approach based on the observation that less conserved glycosylation sites are occasionally absent due to the intrinsic sequence variation in HIV-1. Our results point to the possibility of generating antibodies that target surface features of Env at ‘glycan holes’. We found that binding of anti-CONEs 4 and 5 antibodies to denatured gp120 critically depended on the removal of the proximal glycan. Also, the reactivities of anti-CONEs 1 and 4 antibodies with the SOSIP Env were greatly enhanced after deglycosylation. The magnitude of inhibitory effect upon HIV-1 infectivity was modest with the anti-CONE 1 sera tested, although the consistency of the effect against multiple isolates of HIV-1 Env pseudoviruses is indicative of a robust activity.

Rational protein design has reinvigorated vaccine design efforts^19,31,32^. Scaffolded immunogens can focus immune responses to known neutralizing antibody determinants^31^ or, as in our work with the HIV-1 CONEs, can facilitate the exploitation of new vulnerable sites. The CONEs reside on an immunogenic ‘silent face’ of gp120; however, a recent study reported the identification of neutralizing antibody VRC-PG05 that recognizes several CONE 1 residues (E293, N448) as well as CONE 1 glycans (N295, N448) (Supplementary Fig. 13)^33^. This observation suggests the presence of germline antibody precursors that engage CONE-related epitopes. To achieve a functional broadly neutralizing response, a viable approach could be administrating a cocktail of CONE mimetics and SOSIP Env protein missing corresponding CONE glycans in successive and combinatorial boosts, focusing the response on the ‘glycan hole’. We expect that rational design of small, thermal-stable CONE-like immunogens represents a promising starting point for the development of reproducible vaccines against persisting infections.

## Methods

### Determination of the consensus sequence of subtype C gp120

The consensus amino acid sequence of gp120 subtype C was obtained by aligning 183 full-length *env* sequences derived by single genome amplification^10^. The dataset includes: 68 acute *env* sequences, 65 chronic *env* sequences, 21 functional *env* clone sequences from GAVI study, and 29 *env* sequences from viruses present in a group of participants with low CD4 counts. The amino acid position was considered a consensus if it was present in 80% of the isolates. The consensus sequences illustration for Fig. 1d was prepared using WebLogo^34,35^.

We also modified the original sequence of a soluble gp140 protein (SOSIP BG505.664) so that the exposed amino acid residues of the protein that originally belonged to HIV-1 subtype A virus were replaced with residues from HIV-1 subtype C virus. The modifications were performed for the regions that correspond to CONE 1, 2, 4, and 5. Sequence manipulations were performed using BioEdit software and HIV Sequence Database Tools available at Los Alamos National Laboratory website (https://www.hiv.lanl.gov/content/sequence/HIV/mainpage.html).

### Computational protein design

The identification of scaffold proteins for transplantation of CONE epitopes made use of the *Erebus* substructure search server (https://dokhlab.med.psu.edu/erebus)^25^. Query structures for this server were defined as the backbone atoms (Cα, Ν, C=O) of each individual CONE (β 12/13/22, α2-helix, loop C, and loop E). We aligned the structures of Env trimer (PDB ID: 4TVP and 4NCO for clade A Env, 5FYK for clade B, 5FYJ for clade G) and extracted the coordinates of backbone atoms. The query structures (PDB format) were then provided to *Erebus* with default search parameters (matching precision 0.5 and minimum weight ‘auto’). *Erebus* scans PDB for matches of structural scaffolds to atom pairs in the query. These resulting scaffolds were ranked based on their RMSD to the query structure (Supplementary Table 1).

Redesign of preexisting scaffolds was accomplished using *Eris*, a computational platform that automatically performs side-chain repacking and backbone relaxation and calculates the changes in free energy upon mutations (ΔΔ*G* = Δ*G*_MUT_ − Δ*G*_WT_, https://dokhlab.med.psu.edu/eris)^27,36^. The solvent-exposed area of each residue in Env was computed with Naccess 2.1.1 (ASA value > 0.3 means exposed). The exposed residues within each CONE were introduced into the corresponding scaffolds by *Eris*. For each single mutation, *Eris* typically performed 100–300 calculations to reach a converged distribution of ΔΔ*G* values. The average ΔΔ*G* and its standard deviation were obtained. Highly destabilizing mutations (ΔΔ*G* > 6 kcal mol^−1^) were discarded. The structures of design models were then relaxed and equilibrated by all-atom DMD simulations.

To estimate the structural rigidity of CONE epitopes within designed proteins, MD simulations were performed under physiological conditions (100 ns simulation at 298 K, 150 mM NaCl, and neutral pH) with Gromacs^37^. The force field CHARMM36 was adopted with the explicit solvent model TIP3P^38^. The simulations were performed at constant temperature (V-rescale thermostat) and pressure (1 bar, Parrinello-Rahman NPT ensemble). The non-bonded interaction cut-off for electrostatics calculations was set to 10.0 Å, and the particle mesh Ewald (PME) method was used in the calculation of long-range electrostatic interactions. Three independent simulations were performed for each design, and the trajectories were analyzed to derive information about the average structure and RMSF.

### Protein expression and purification

The genes encoding the designed proteins were synthesized by Biomatik and cloned into BamHI and NcoI restriction sites of pET14b vectors (Invitrogen). All constructs were confirmed by DNA sequencing. *E. coli* BL21(DE3) pLysE strains transformed with each individual gene were grown to an optimal density (optical density 0.6 at 600 nm) in Lysogeny broth, and then gene expression was induced with 1 mM isopropyl-β-d-thiogalactopyransoide. Proteins expressed with an N-terminal His tag (MGHHHHHHGSENLYFQG) were purified on a HisTrap column (GE Healthcare) and then by size exclusion chromatography (HiPrep 16/60 Sephacryl S-200 HR). Briefly, cell pellets were resuspended in lysis buffer (20 mM NaH_2_PO_4_/Na_2_HPO_4_, 40 mM imidazol, 100 mM NaCl, 5 mM β-mercaptoethanol, pH 7.4) with protease inhibitors (phenylmethylsulfonyl fluoride and pepstatin A) and lysed by sonication. The supernatant containing protein components was separated from precipitate by centrifugation at 24,000 × *g* for 30 min, passed through 0.22-μm filter (Millipore), and then loaded onto a HisTrap column. Proteins were eluted with a gradient (5–90%, 500 mM imidazol as 100%) of imidazol in 20 mM NaH_2_PO_4_/Na_2_HPO_4_, 100 mM NaCl, pH 7.4. For the Sephacryl 200 column 50 mM NaH_2_PO_4_/Na_2_HPO_4_ and 150 mM NaCl (pH 7.4) was used as running buffer. The His tag was removed by TEV protease (Sigma-Aldrich) following the manufacturer’s procedure. The cleaved proteins were enriched in flow through of the HisTrap column and were further purified through a HiLoad 16/600 Superdex 200 preparatory-grade column (GE Healthcare). The His tags were not removed from proteins conjugated to nanoparticles for immunization.

For NMR measurements, *E. coli* BL21(DE3) pLysE strains containing the C1S1 gene (in pET14b) were grown in M9 minimal medium with 1.0 g L^−1 15^NH_4_Cl and 2.0 g L^−1 13^C dextrose (Cambridge Isotope Laboratories, Inc.). Proteins were expressed and purified following the same procedure as used for those obtained from cells grown in Lysogeny broth medium.

Chemicals and enzymes were purchased from Sigma-Aldrich unless otherwise indicated. Protein concentrations were determined on a NanoDrop spectrophotometer using absorption at 280 nm (Thermo Scientific).

### Biophysical characterization

CD spectroscopy data was collected using a Chirascan Plus instrument (Applied Photophysics, MA). The protein samples were dialyzed against 50 mM phosphate buffer (pH 7.4) and diluted to 0.2 mg mL^−1^ before analysis. CD spectra were measured from 260 to 185 nm at 20 °C with 50,000 readings taken with 0.5-nm increment. Melting curves were monitored from 20 to 94 °C at 220 nm and fitted to a two-state folding model for the estimation of melting temperatures^39^.

The protein samples for size exclusion analysis (10 μM protein in 40 mM Na_2_HPO_4_/NaH_2_PO_4_, 100 mM NaCl, pH 7.4) were loaded onto a Superdex 200 10/300 GL column (GE Healthcare, PA) and eluted with the same PBS buffer at a flow rate of 0.4 mL/min. UV absorption at 280 nm was monitored. Three molecular weight markers were also analyzed: cytochrome C (12.4 kDa), aprotinin (6512 Da), and vitamin B12 (1355 Da). The log of molecular weight versus elution volume for those proteins was plotted for the column and used to calculate the apparent molecular weight of designed proteins.

### Crystallization, data collection, phasing, and refinement

Protein samples for crystallization (10–50 mg mL^−1^ in 20 mM Tris, 150 mM NaCl, pH 8.0) were prepared by column chromatography and ultrafiltration. Sparse matrix screens in 96-well sitting drops were performed on Rigaku Phoenix Liquid Handler with MCSG Crystallization Suite. Crystallization conditions were then optimized for C2S5 and C4S3 in 24-well hanging drops. C2S5 was crystallized in 16% (w/v) polyethylene glycol 20000, 100 mM MES, pH 6.5. C4S3 was crystallized in 30% (w/v) polyethylene glycol 3350, 200 mM Bis-tris pH 6.5, and 200 mM LiSO_4_. Crystals were cryoprotected in the reservoir solution supplemented with 10% (v/v) glycerol, then flash frozen and stored in liquid nitrogen. The crystals were checked for high quality X-ray diffraction using Rigaku Saturn 944 + CDD with ACTOR sample changing robot. Diffraction data was then collected on Advanced Photon Source (Argonne National Laboratory) at beamlines 22-ID and 22-BM (wavelength 0.9782 Å) and processed on HKL2000^40^. The structures of C2S5 and C4S3 were solved by molecular replacement with Phaser of CCP4i and PHENIX^41,42^. Briefly, the crystal structures of the scaffold proteins (PDB ID 1BZ4 for C2S5 and 2W4C for C4S3) were used as search models. The structures were built and manually adjusted in Coot and then refined by Refmac5 (or by PHENIX with Composite omit map and TLS refinement options). The structures were validated using PDB validation server, Molprobity and Chiron server (https://dokhlab.med.psu.edu/chiron)^26,43^. Data collection and final refinement statistics are shown in Table 1.

### NMR

For NMR measurements, ^13^C and ^15^N-enriched C1S1 protein (1 mM) was exchanged into NMR buffer (20 mM Na_2_HPO_4_/NaH_2_PO_4_ pH 6.0). Five percent (v/v) D_2_O and 10 μM DSS (4,4-dimethyl-4-silapentane-1-sulfonic acid) were added to the protein solution. NMR spectra were acquired at 25 °C on a Bruker Avance III 850 NMR spectrometer. 2D ^1^H-^15^N HSQC experiments were recorded using 16 scans per increment and a recovery delay of 1.0 s with 2048 and 256 complex points in the direct and indirect dimensions, respectively. Spectral widths used were 7911.393 Hz (^1^H) and 2152.949 (^15^N) Hz. Average ^1^H-^15^N chemical shift perturbations were calculated according to the square root of ((Δ*σ* ^1^H)^2^ + (Δ*σ* ^15^N)^2^/25), where Δ*σ* ^1^H and Δ*σ* ^15^N are the observed changes in ^1^H and ^15^N chemical shifts.

^1^H-^15^N HSQC spectra allow for the detection of protons directly bonded to a ^15^N, including both backbone and side-chain NH resonances. An NH resonance can be detected for every residue except for proline and the spectrum contains a ‘fingerprint’ of the protein backbone. Backbone resonance assignments of C1S1 were obtained by analysis of HNCACB and CBCA(CO)NH spectra recorded on ^13^C and ^15^N-labeled C1S1 at the same concentration employed for 2D HSQC experiments. The spectra were recorded with 2048 (^1^H), 64 (^15^N), and 128 (^13^C) complex points. Spectral widths were 10204.082 Hz (^1^H), 2929.115 Hz (^15^N), and 12820.513 Hz (^13^C).

The assignments of Cα, Cβ, N, and HN chemical shifts were performed with an iterative procedure using the program MARS and manual inspection^44^. For chemical shift indexing (CSI), ΔCα and ΔCβ values were calculated by subtracting experiment chemical shifts of Cα and Cβ from random coil values obtained from the ncIDP server^45^. The value of ΔCα−ΔCβ was calculated to cancel the systematic offset contained in ΔCα and ΔCβ and then used to predict secondary structure. G17, G25, G43, G57, G61, G63, and G86 have no Cβ and their ΔCα−ΔCβ values were not calculated. Spectra were processed and analyzed using NMRPipe (NIDDK, National Institutes of Health) and Sparky (University of California, San Francisco).

### General immunization procedure

Immunogen injections and animal handling were performed by the Division of Laboratory Animal Medicine staff of UNC-Chapel Hill. All work with animals followed protocols that were approved by the UNC Chapel Hill Institutional Animal Care and Use Committee. Intra-muscular injections were performed on 8–12-week-old New Zealand white female rabbits (2.5–3.0 kg) obtained from Robinson Services. The immunization protocols are summarized in Supplementary Table 3. The immunization scheme was an initial dose of 300 μg of each CONE followed by boost injections, supplemented with either incomplete Freund’s adjuvant according to the standard protocol, or nickel-conjugated nanoparticles without additional adjuvant. Immunogen boost injections with the same amount of protein were performed 3 weeks apart. The immunization of loop C peptide was performed by the Custom Antibodies department of Thermo Fisher Scientific company.

For CONE 2 immunization, two changes were introduced. First, the single scaffold-immunogen (C2S5) was compared to two scaffold-immunogens (C2S5 + C2S3) approach. In the latter approach, we alternated injections of the proteins with the same structure and HIV-specific sequence presented on different scaffolds. Second, we assessed the possibility to use the saponin-based adjuvant Matrix-M™ (Novavax AB) as a substitute for the nanoparticles. Matrix-M adjuvant was kindly provided by Novavax, Inc. For the first 8 weeks, 30 µg of protein were given at each injection. Lower antibody titers were observed than previously obtained with 300 μg of conjugated protein; therefore, the CONE 2 immunization plan was amended. After 8 weeks the amount of immunogen was increased from initial 30 μg protein to 300 μg conjugated on nanoparticles, without an adjuvant. This change of protein amount is noted in Fig. 3a.

### Immunization with nanoparticles

Nickel nanoparticles (NPs) were prepared from warm oil/water (o/w) microemulsion precursors following a reported procedure^46,47^. In the optimized formulation, Brij 78 (1.75 mg), Brij 78-NTA-Ni (1.75 mg), TPGS (1.5 mg), and Miglyol 812 (2.5 mg) were weighed into a 7 mL glass vial and heated in a water bath to 65 °C to melt and blend all excipients. A small amount of ethanol (100 μL) was added to the melted excipients and the solution was swirled to result in a homogenous mixture. The ethanol was removed completely using a stream of nitrogen and the vial was transferred to a water bath at 65 °C. To the mixture of melted oil and surfactants was added 1 mL of filtered and deionized water pre-heated at 65 °C, and the solution was stirred for 30 min at 65 °C then cooled to room temperature.

NPs (1 mL batches, *n* = 3 for each protein concentration) were characterized for particle size (NanoSight), zeta potential, and Ni content Ni content (ICP-MS) prior to addition of protein. The Ni-NPs (0.5 mL) were incubated with the corresponding amount of His-tagged protein (C1S1, C2S5, C4S3, or C5S3) at 4 °C overnight. Final protein concentrations added to 0.5 mL of Ni-NPs were: 150 μg mL^−1^, 240 μg mL^−1^, 300 μg mL^−1^, 400 μg mL^−1^, and 500 μg mL^−1^. Free His-tagged protein was removed by spin filtration using VIVASPIN 500 ultrafiltration tubes (300 kDa MWCO). Sample containing protein and NPs was transferred to a spin filtration tube and spun at 16,000 rcf for 30 min to remove free unconjugated protein. Purified samples were analyzed by UV absorbance at 280 nm before and after spin filtration to quantify the percent conjugation of His-tagged protein to NPs at each concentration (*n* = 3). The UV absorbance was measured using a BioTek Synergy 2 UV Spectrometer (Winooski) at a wavelength of 280 nm.

Polyoxyethylene (20) stearyl ether (Brij 78) was purchased from Uniqema (Wilmington, DE). D-alpha-tocopheryl polyethylene glycol 1000 succinate (TPGS) was purchased from Eastman Chemicals (Kingsport, TN). Miglyol 812 is a mixed caprylic (C8:0) and capric (C10:0) fatty acid triglyceride and was purchased from Sasol (Witten, Germany). Brij 78-NTA-Ni was synthesized by Dr. Benhabbour using a reported procedure^46^. Acetonitrile (CH_3_CN), dichloromethane (CH_2_Cl_2_), and ethanol (EtOH) were purchased from Fisher Scientific.

### Particle size and zeta potential

NPs samples were run on a NanoSight NS500. All samples were diluted to a concentration between 1 × 10^8^ and 5 × 10^8^ particles per mL in deionized H_2_O. Five 60-s videos were taken of each sample to capture particles movement. The NanoSight software tracked the particles individually and using the Stokes–Einstein equation, calculated the hydrodynamic diameters. The zeta potential of NPs was measure in PBS (pH 7.4) using a Malvern Zeta Sizer 2000 (Malvern Instruments).

### ICP-MS analysis

Nickel content was quantified by inductively coupled plasma mass spectrometry (ICP-MS). The Agilent 7500cx ICPMS is outfitted with an octapole reaction cell (ORC) and a high matrix introduction (HMI) system. Standard operating conditions were RF Power 1550 W, argon flows and plasma gas flow 15 L min^−1^, carrier gas flow 1.03 L min^−1^, makeup gas 0.15 L min^−1^ and sampling depth was 8.0 mm. All solutions were prepared using 18 MΩ deionized water and trace metal grade nitric acid (SCP Science). The instrument was tuned daily to maximize sensitivity and minimize production of oxides and doubly charged ions. Sample flow rate was 330 µL min^−1^ through a Mienhard TRP-50-A0.5 nebulizer and the Scott double pass spray chamber was cooled to 2 °C. Helium flowed at 4 mL min^−1^ through the ORC to eliminate isobaric interferences, ^44^Ca^16^O^+^ and ^23^Na^37^Cl^+^, of ^60^Ni. Standards were prepared using single element standards purchased from High Purity Standards. Scandium was used as internal standard and introduced continuously through a tee junction. Ions ^45^Sc (internal standard) and ^60^Ni were monitored in a peak hopping mode, using a 100-ms dwell time, and eight replicates were measured. The Ni standard curve included 13 concentrations levels in the range of 0.5–1000 ppb. This spanned the concentration range of all samples. The standard dataset was fitted to a linear curve. The coefficient of correlation was 0.999. Percent error in calculated concentrations was <5%. For quantitation of Ni in the NPs, preparation included removal of the water from the NPs and resuspension in 2% HNO_3_ solution.

### TEM imaging

Preparations of NPs, Matrix-M adjuvant, and protein alone and in combination were negatively stained with 2% sodium phosphotungstate, pH 7.0. Five microliters of suspension was applied to a glow-discharged formvar/carbon-coated 400 mesh copper grid and allowed to adsorb for 1 min or 5 min depending on concentration. Grids were briefly floated on droplets of deionized water twice to remove buffer salts and were then transferred to a 25-µl droplet of 2% sodium phosphotungstate, pH 7.0 for 30 s. Excess stain was removed by blotting with filter paper and the grids were air dried. Grids were observed with a JEOL JEM-1230 transmission electron microscope (JEOL USA) operating at 80 kV, and digital images were acquired using a Gatan Orius SC1000 CCD camera and Gatan Microscopy Suite 3.0 software (Gatan, Inc.).

### ELISA

The autologous and heterologous binding properties of antibodies to the CONE immunogens and SOSIP Env were tested using the Protein Detector Peroxidase ELISA Kit, Anti-Rabbit IgG (KPL/Sera Care Life Sciences). The ELISA assay was performed according to the manufacturer’s protocol; minor modifications included plate coating overnight at 4 °C and color development for 1 h before addition of a stop solution. Absorbance levels were measured at 405 nm. CONE immunogens coated on microwell plates were exposed to serial dilutions (1:200 to 1:256,000) of the rabbit sera and the lowest titer was determined at the absorbance level >0.4, which was the two times recommended maximum background value for this kit. The primary immunogen and an alternative scaffold were used to confirm the specificity of the immune response. For the SOSIP ELISA assay, the modified gp140 SOSIP-C protein was used. The protein was produced in 293F cell line at the UNC Protein Expression & Purification Core Facility and deglycosylated in native conditions by incubation for 24 h with PNGase F according to the manufacturer’s protocol (New England Biolabs).

### Western blot

Total rabbit IgG was purified from each animal using rProtein A GraviTrap kit (GE) according to the kit protocol, dialyzed against PBS buffer, and diluted to equal amount per CONE immunization group. The gp120 proteins were deglycosylated using PNGase F in SDS buffer (New England Biolabs). Ten percent polyacrylamide gels and standard western blot protocol with SuperBock blocking reagent (ThermoFisher) was applied. The modified gp140 SOSIP-C proteins were produced at UNC Protein Expression & Purification Core. The amount of any protein loaded per lane was 400 ng. Purified IgG pools from immunized animals (1 mg ml^−1^) were used as primary antibodies at a dilution of 1:1500. HRP goat anti-rabbit antibody (Thermo Fisher Cat #32460) was used as secondary antibody at a dilution 1:12500. The fluorescence signal was detected by Amersham ECL Western Blotting Detection Kit (GE) using a BioRad Chemidoc imager. PageRuler Plus Prestained Ladder was used for band size discrimination. To confirm the successful transfer of the gp120 proteins, the blots were stripped using Restore Western Blot Stripping Buffer (ThermoFisher) and restained with anti-gp140 rabbit IgG solution (1 mg ml^−1^ and 1:12,500 dilution) that targeted a variety of epitopes on gp120 and served as our positive control for both glycosylated and deglycosylated proteins. Scans of all blots were supplied in the Source Data file.

### Neutralization assays

To assess neutralizing properties of raised antibodies, we used a panel of HIV-1 pseudoviruses that expressed isolate-specific Env proteins, incubated the virus with rabbit serum, and assessed viral infectivity in the luciferase-expressing TZM-bl cells using a standard protocol (https://www.hiv.lanl.gov/content/nab-reference-strains/html/Protocol-for-Neutralizing-Antibody-Assay-for-HIV-1-in-TZMbl-cells_Apr2017.pdf). TZM-bl cells were obtained from NIH AIDS Reagent program (cat. # 8129). Based on similarities to CONE immunogens, we chose the following HIV-1 isolates with corresponding GenBank IDs: 0665 (GB# KC894076), 0682 (GB# KC894077), 1086 (GB# KC894079), 3003 (GB# KC894087), and 3037 (GB# KC894098). In those cases where the natural isolate lacked one or more of the relatively conserved glycosylation sites, these were added back to create the fully glycosylated form for that isolate. The fully glycosylated form was then mutated to remove the glycosylation site at Env positions specific for CONE proteins. For CONE 1, a glycan at the position 332 (N332S) or at the position 448 (N448S) was removed. For CONE 2, a single glycan in alpha-helix at the position 337 (N337/339S) was removed. For CONEs 4 and 5, the glycans N289S and N356/358S were removed, respectively. All positions are listed in reference to HXB2 HIV-1 viral strain. The envelope sequences of the isolates were pseudotyped on the HIV-1 subtype C viral backbone, and single-cycle infection virus was produced in HEK293T cells. Pre- and post-immunization sera were heat inactivated and diluted 1:10 in cell growth media. The inhibitory properties of post-immunization serum were compared to those of the pre-immunization serum for each animal. Data were analyzed and plotted using GraphPad Prism 6.0 software.

### Reporting summary

Further information on experimental design is available in the Nature Research Reporting Summary linked to this article.

## Supplementary information

Supplementary Information

Reporting Summary

## Data Availability

The source data underlying Figs 2c, 3, 4 and Supplementary Figs 6, 7, 8, 9, 11 and 12 are provided as a Supplementary Source Data file. The coordinates of the designs C2S5 and C4S3 are available from the RCSB Protein Data Bank with the accession codes 6CFE and 6CBU. A reporting summary for this Article is available as a Supplementary Information file. All other data supporting the findings of this manuscript are available from the corresponding authors (N.V.D. and R.S.) upon reasonable request.
