# Supplementary material for: Rationally designed carbohydrate-occluded epitopes elicit HIV-1 Env-specific antibodies
Source: Nat Commun. 2019 Feb 27;10:948. doi: 10.1038/s41467-019-08876-w (PMC6393580; doi:10.1038/s41467-019-08876-w)
Supplement: Supplementary file 1 — Supplementary Information [file 41467_2019_8876_MOESM1_ESM.pdf]

Rationally Designed Carbohydrate-Occluded Epitopes Elicit HIV-1 Env-Specific Antibodies

Zhu *et al*.

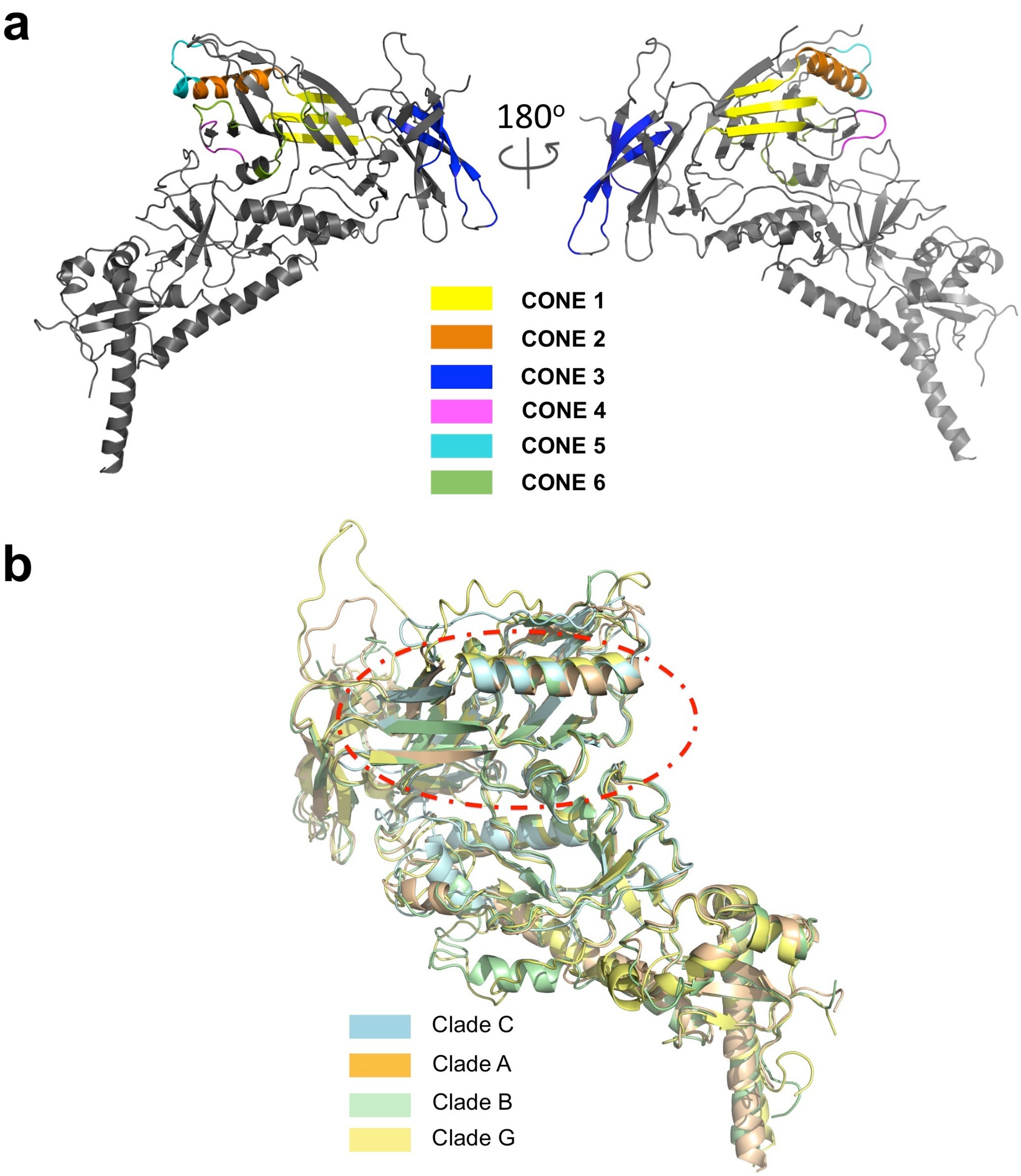

Supplementary Figure 1. Overview of the CONE structures. a, Six structural elements identified as CONEs in the Env protomer (PDB ID 5FYL). The gp120/gp41 interface was not shown in the structural model. **b,** Superposition of gp120 structures from four HIV-1 clades (PDB 5FYL for clade A Env, 5FYK for clade B, 3TGR for clade C, 5FYJ for clade G). The outer domain regions containing the CONEs are within the region highlighted by red ellipse.

ß12_CONE 1	V	E	I	V	C	T
	V153	E100 (55%)	I183	V95 (52%)	C183	T137 (75%)
	I28	K20		N31		I26
	L2	Q16		E25		A7
		G12		T19		V7
		N10		M4		M3
		A7		I3		R2
		D7		K2		K1
		P4		L2		
		R4		Q1		
		S1		S1		
		T1				
		V1				

ß13_CONE 1	H	C	N	I	S
	H141 (77%)	C183	N157 (86%)	I168	S128 (70%)
	Y40		T8	V12	N40
	S2		K5	L3	T11
			D4		D2
			S3		K1
			E2		R1
			I2		
			H1		
			Q1		

ß22_CONE 1	N	I	T	C	K	S	N
	N153 (84%)	I178	T169 (92%)	C183	K61 (33%)	S182	N130 (71%)
	S10	V3	I7		N48	A1	S36
	K8	L2	A4		R22		T7
	E3		R1		E16		E3
	D2		S1		T16		K3
	I2		V1		I11		D2
	T2				S5		I1
	A1				V3		Y1
	L1				D1		
	V1						

α2 Helix_CONE 2	K	S	K	W	N	K	T	L	Q	R	V	G	E	K	L
	K63 (34%)	S27 (15%)	K57 (31%)	W183	N133 (73%)	K66 (36%)	T168	L175	Q78 (43%)	R53 (29%)	V173	G46	E73 (40%)	K164	L181
	E48	E26	N25		T17	E35	A9	V5	E45	K43	I10	K44	K63	E7	F2
	G20	K25	D20		S10	R24	I3	I3	K17	Q27		S43	R18	R6	
	R18	T24	A19		D7	N18	M2		N8	E19		R20	D6	Q3	
	A15	G22	Q17		E7	T18	L1		Y8	G13		A7	G6	T2	
	T7	A14	E15		H4	Q5			R7	N9		V7	Q5	S1	
	L2	D14	L6		G2	D5			L5	W8		Q5	T4		
	N2	Q11	R5		Y2	A4			G4	M5		E4	S3		
	Q2	N6	T5		I1	G3			I4	D3		T3	I2		
	D1	R5	G4			S3			H3	A1		I2	N2		
	I1	V3	S4			I1			A2	L1		N1	A1		
	S1	L1	V4			Y1			D1	S1		H1			
	W1	H1	--2						S1						
	--2	F1													
		W1													
		--2													

Loop C_CONE 4	L	A	E	E	E	I
	L174 (95%)	A178	E170 (93%)	E104 (57%)	E138 (75%)	I171
	!7	S4	K8	G42	G20	V9
	T1	V1	G2	K28	D17	T3
	R1		D1	Q5	K6	
			--2	R2	N1	
				N1	R1	
				--1		

Loop E_CONE 5	K	E	H	F	P	--	N	K	T
	K55 (30%)	E130 (71%)	H132 (72%)	F169	P126 (69%)	K1	N154 (84%)	K146 (80%)	T147 (80%)
	R37	K35	Y29	Y10	N8	N1	G7	R13	N17
	Q22	G6	L13	L2	S8	--181	D6	T6	E5
	A20	T4	Q3	I1	L7		S6	S4	K4
	E16	N2	R2	S1	H6		K5	G3	S4
	G7	R2	C1		T2		E2	N3	A2
	S7	V2	E1		A1		A1	--2	P2
	L6	A1	F1		D1		P1	A1	D1
	T5	I1	S1		R1		--1	D1	I1
	H2				E1			E1	
	N2				-22			I1	
	V2							M1	
	Y2							Q1	

Supplementary Figure 2. Protein sequence variances of CONEs 1, 2, 4, and 5. The numbers in parentheses indicate the sequence conservation rate of exposed CONE residues in the consensus sequence.

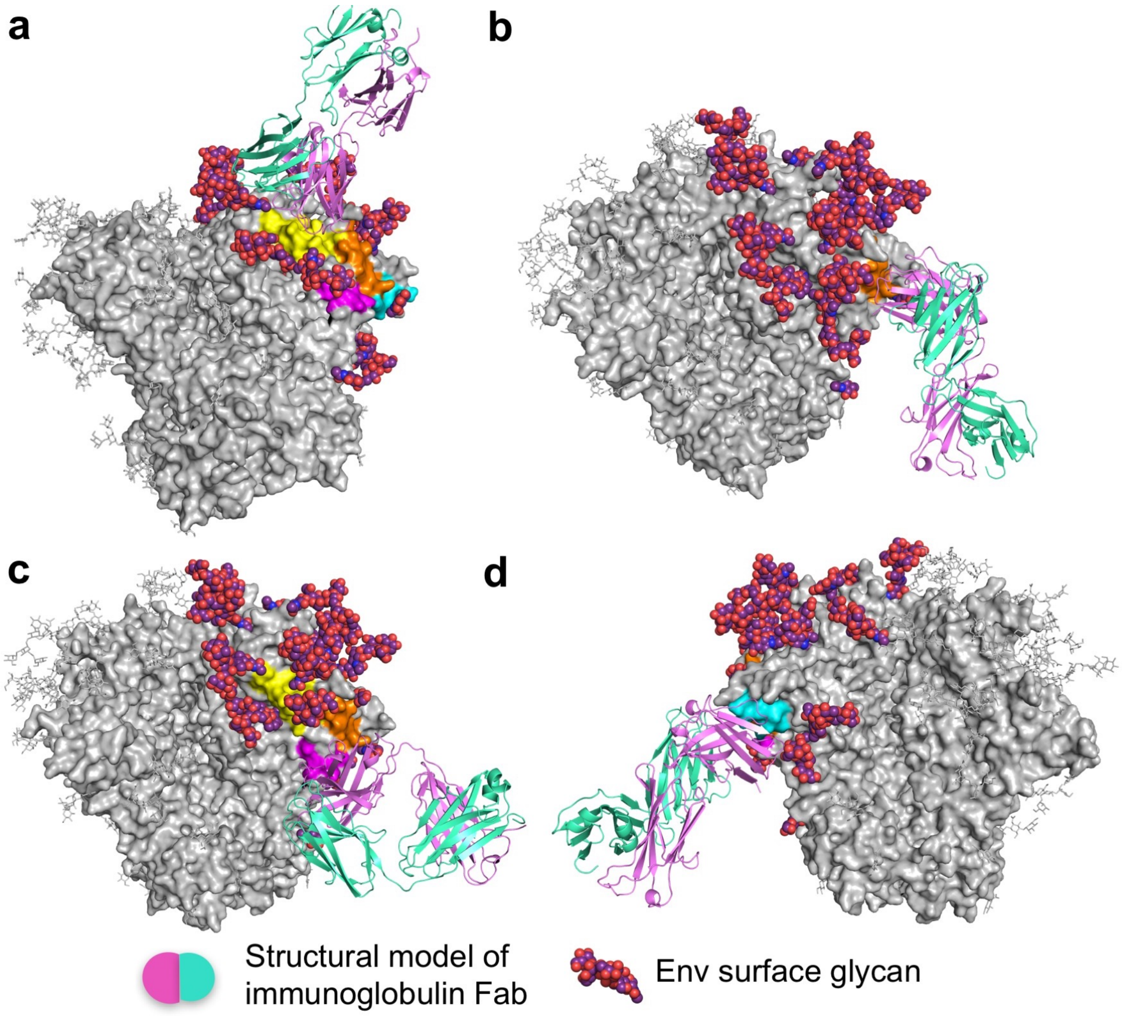

Supplementary Figure 3. Antibody recognition of the CONEs in computational models. CONEs are associated with one or more specific glycosylation sites. The absence of these carbohydrates as the natural course of viral sequence variation exposes the underlying structure, which can be targeted by antibodies: **a,** CONE 1; **b,** CONE 2; **c,** CONE 4; **d,** CONE 5. In the docking models of 'pseudo-antibody' antigen-binding fragment (Fab) and Env (grey surface), the glycans are shown as space-filling spheres and Fabs are shown as cartoon ribbons. CONEs are highlighted with the same color pattern as in Fig.1 of the main text.

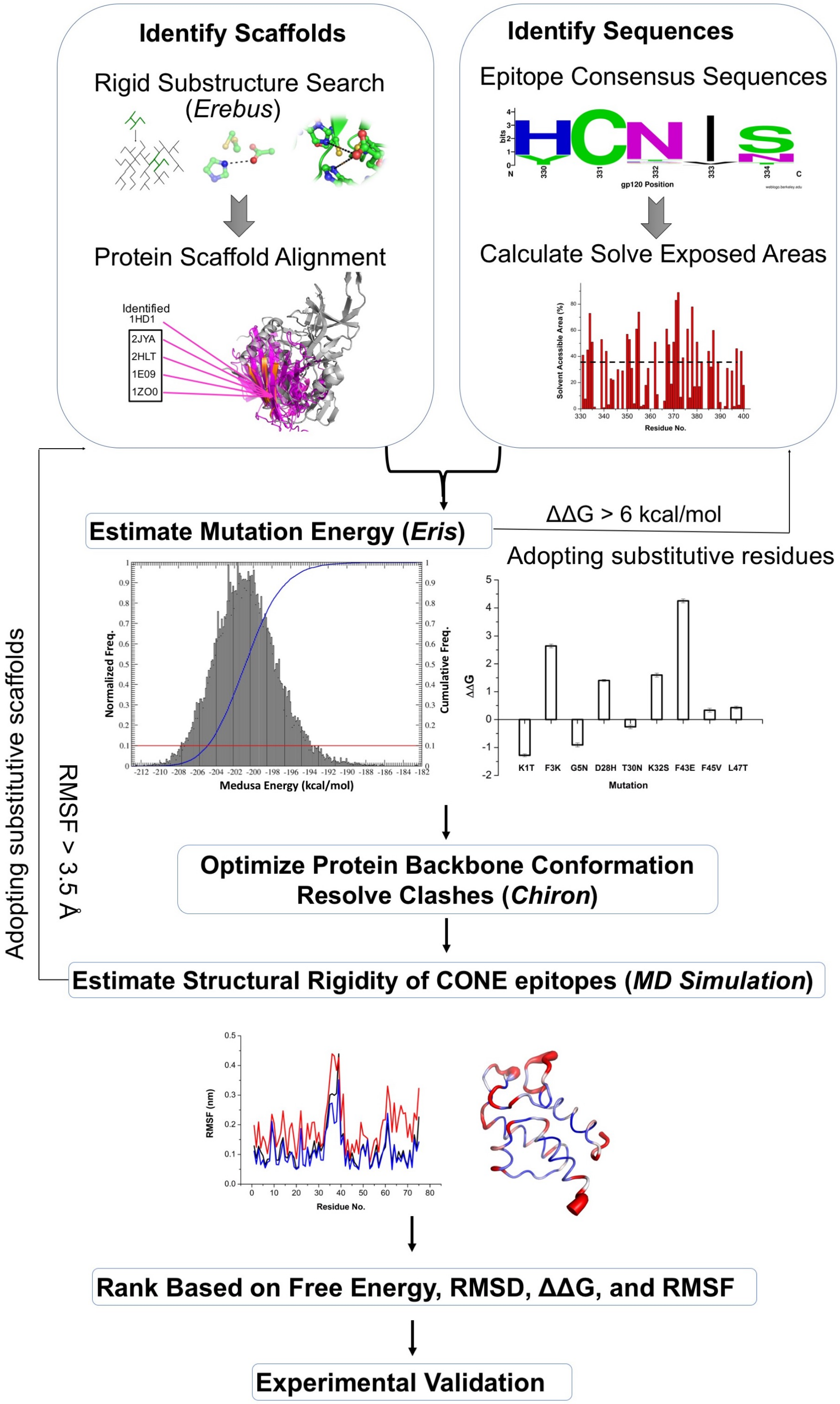

Supplementary Figure 4. Flow chart illustrating the key steps in the design of CONE immunogens. Representative outcome of each step was shown.

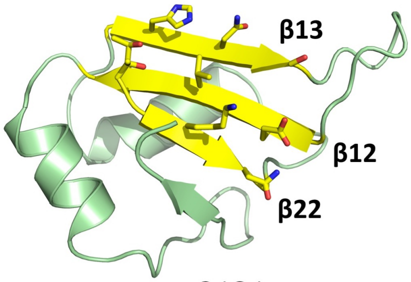

C1S1

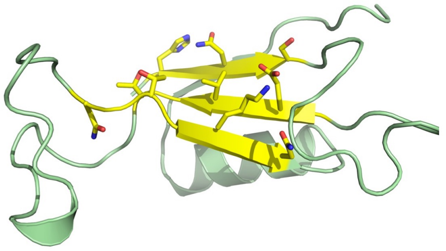

C1S2

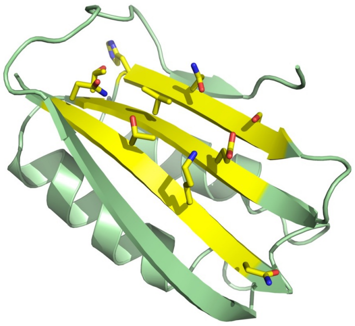

C1S3

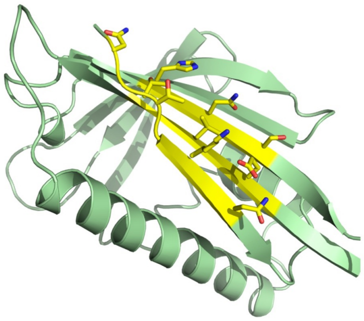

C1S4

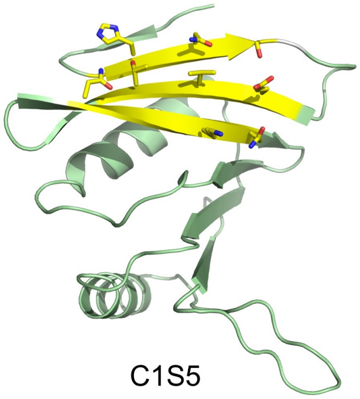

C1S5

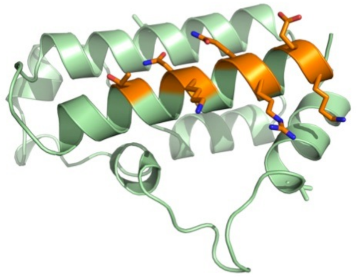

C2S1

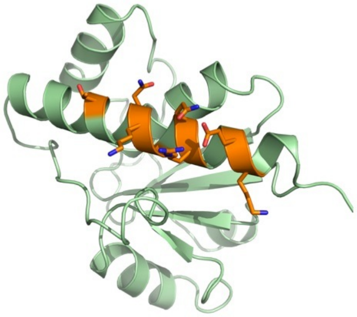

C2S2

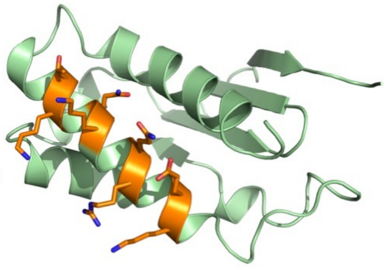

C2S3

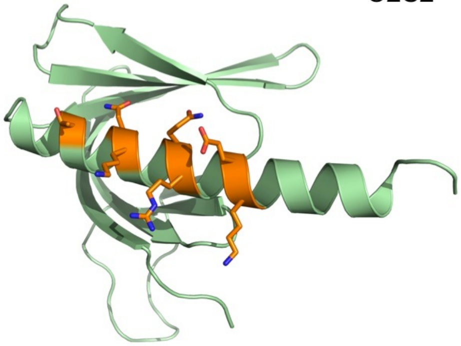

C2S4

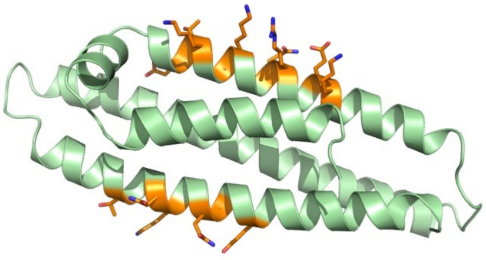

C2S5

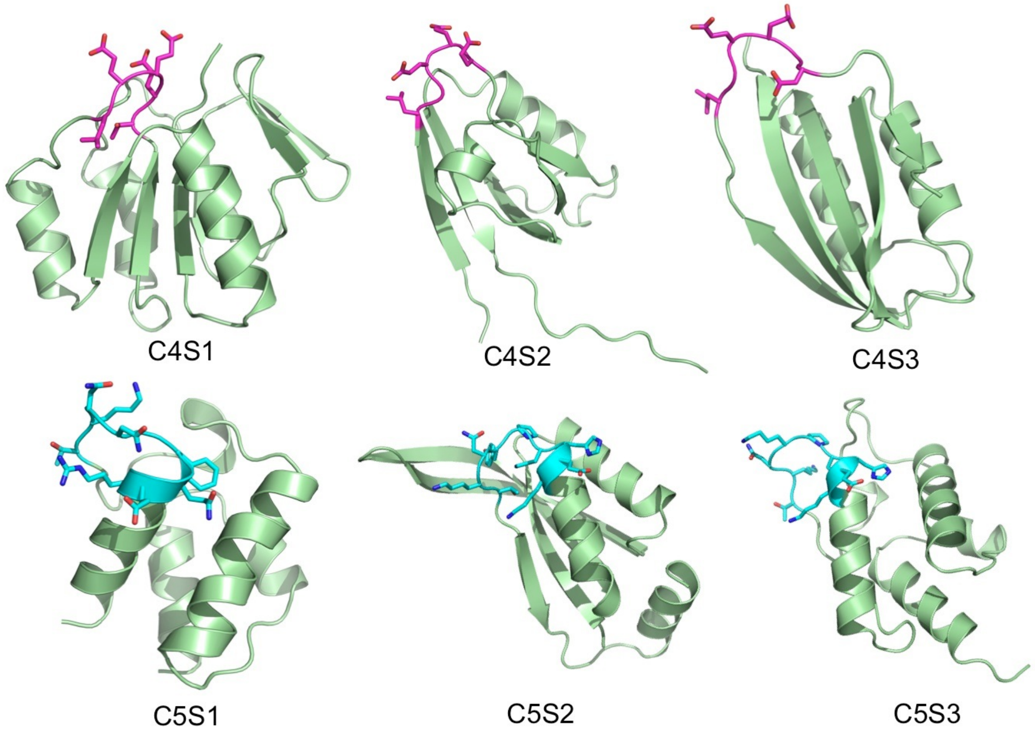

Supplementary Figure 5. Computational design models that present CONEs in heterologous lightweight protein scaffolds. Five designs were shown for CONEs 1 and 2, and three designs were shown for CONEs 4 and 5, adopting the consensus sequences shown in Fig. 1d. CONEs are highlighted with the same color pattern as in Fig.1. The β 12/13/22 strands were labeled on C1S1 and similar arrangements of three strands were adopted in other CONE 1 designs.

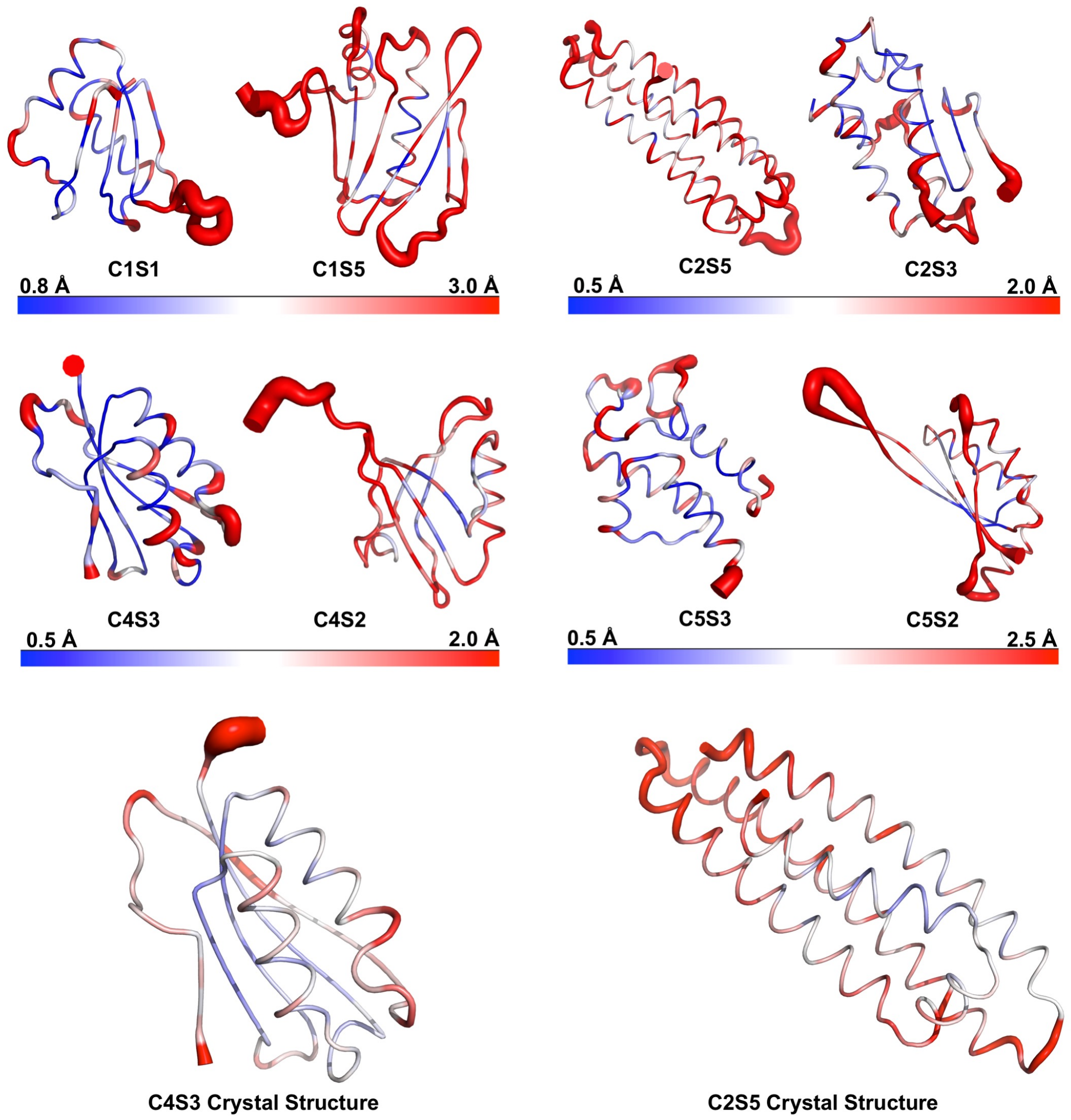

Supplementary Figure 6. The conformational plasticity of each design assessed with RMSF (for computational model) and B factor (for crystal structure). For each CONE, the primary designs (C1S1, C2S5, C4S3, and C5S3; used for immunization) exhibited higher structural rigidity (lower RMSF) in comparison to the alternative designs (C1S5, C2S3, C4S2, and C5S2). The similarity between the crystal structure B factors (PDB 6CBU for C4S3 and 6CFE for C2S5) and the simulated RMSF of corresponding models demonstrated that molecular dynamics could predict the relative structural rigidity of grafted epitopes. Source data are provided as a Source Data file.

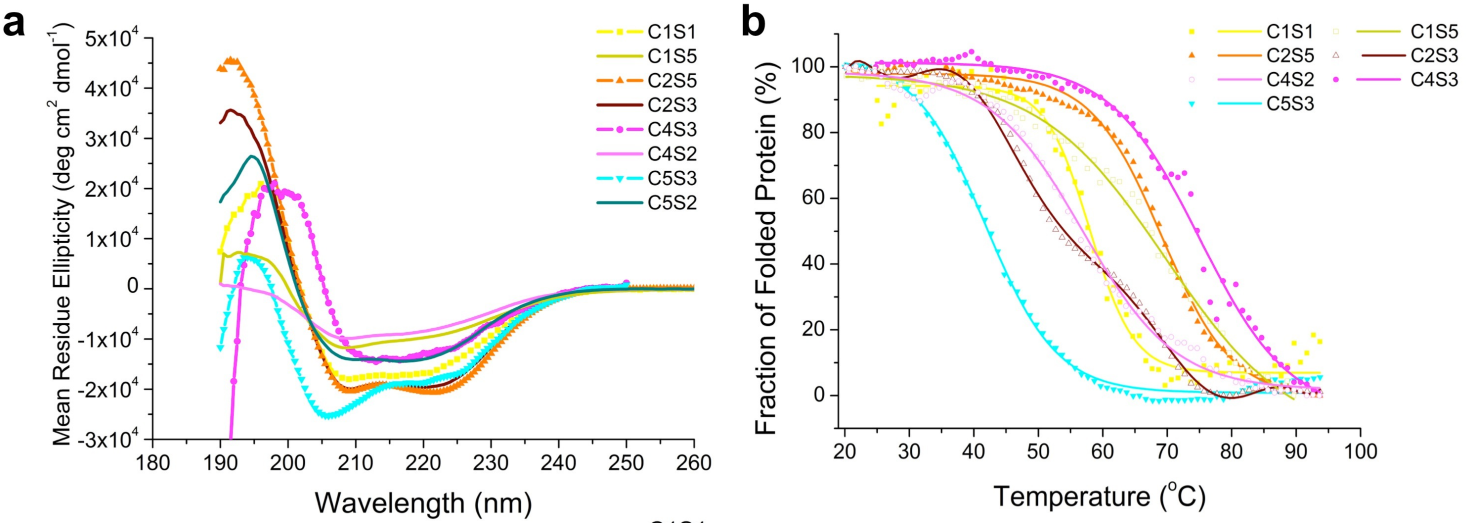

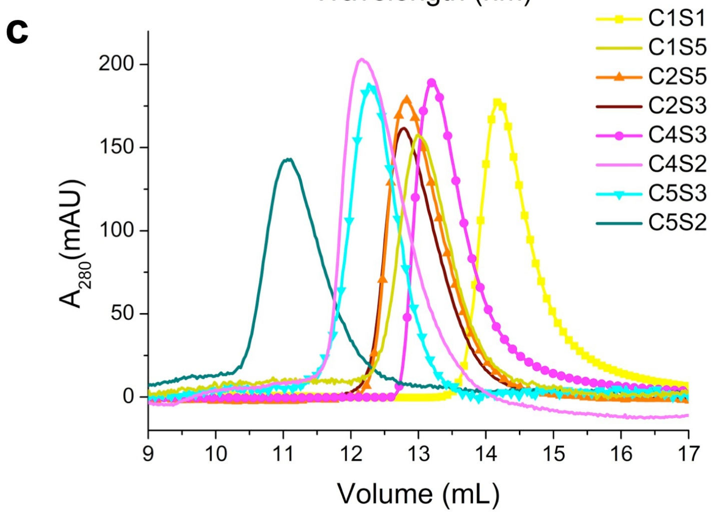

d

Protein ID	Molecular Weight / kD	Calculated Molecular Weight /kD	Aggregation States
C1S1	8.70	8.51	0.98
C1S5	14.30	13.02	0.91
C2S5	16.79	14.10	0.84
C2S3	14.44	13.86	0.96
C4S3	11.20	11.74	1.05
C4S2	10.20	13.88	1.36
C5S3	12.09	15.96	1.32
C5S2	12.07	22.81	1.89

Supplementary Figure 7. Biophysical characterization of the designed CONE-immunogens. a, The CD spectra of eight designs indicated that expected secondary structures were adopted in solution. **b,** The denaturation curves obtained by plotting % folded protein (based on the molar ellipticity at 220 nm) of six designs were indicative of two-state transitions (C2S3 featured a three-state transitions) and properly folded protein structures in solution. **c,** Analytical SEC revealed that seven designs folded into monomers; C5S2 formed a dimer in solution. **d,** Table lists actual molecular weights and those calculated based on SEC. An aggregation state of 1 is indicative of a monomer and 2 indicates a dimeric state. Source data are provided as a Source Data file.

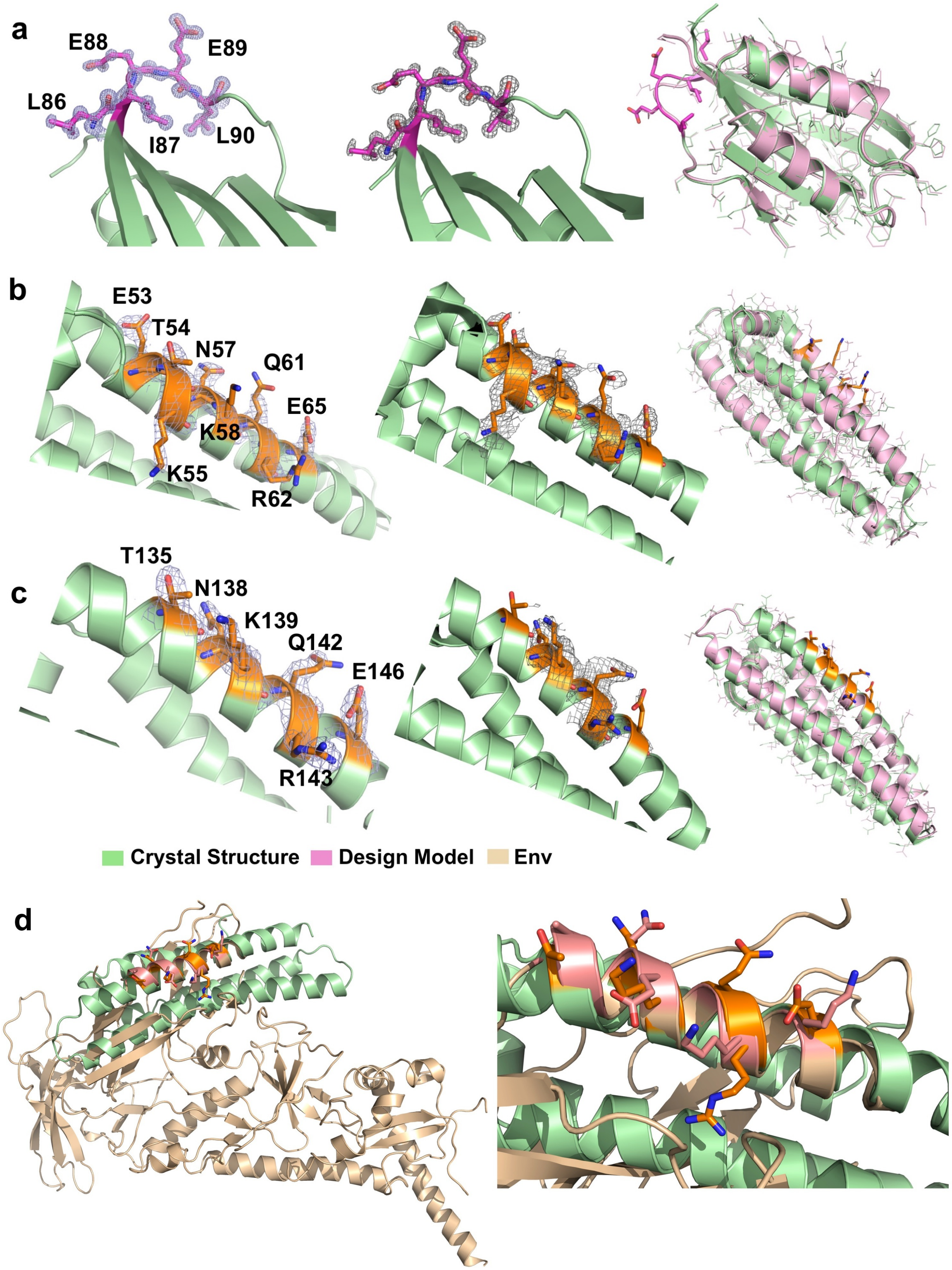
a
E88
E89
L86
I87
L90
b
E53
T54
N57
Q61
E65
K58
K55
R62
c
T135
N138
K139
Q142
E146
R143
Crystal Structure
Design Model
Env
d

Supplementary Figure 8. The conformations of CONE 2 and CONE 4 epitopes in crystal structures. a, (Left) 2Fo-Fc electron density of grafted loop C in C4S3 (resolution 1.2 Å, contoured at 2.0 σ). (Middle) To further validate the conformation of CONE epitopes and reduce possible structural bias, the coordinates of grafted epitopes (two segments of α-helix for C2S5 and loop C for C4S3) were deleted in the initial structural model of refinement. The Fo-Fc omit map (contoured at 3.0 σ) of resolved epitopes was shown as a comparison to the 2Fo-Fc map. (Right) C4S3 crystal structure aligned to the design model (pink). The CONE-residues were shown as sticks (magenta) and other residues were shown as lines. C4S3 possesses two intertwining βαβ motifs and the high resolution of C4S3 crystal structure allowed us to unambiguously determine the conformation of grafted loop C. **b-c, (**Left) 2Fo-Fc map of grafted α2 helix in two different segments of C2S5 (resolution 2.0 Å, contoured at 1.3 σ). (Middle) Fo-Fc omit map (refined as described for C4S3, contoured at 2.0 σ). (Right) C2S5 crystal structure aligned to design model (pink). The CONE-residues were shown as sticks (orange) and other residues were shown as lines. **d,** The second grafted α2 helix aligned to gp120 CONE 2 region (PDB 5FYL). Source data are provided as a Source Data file.

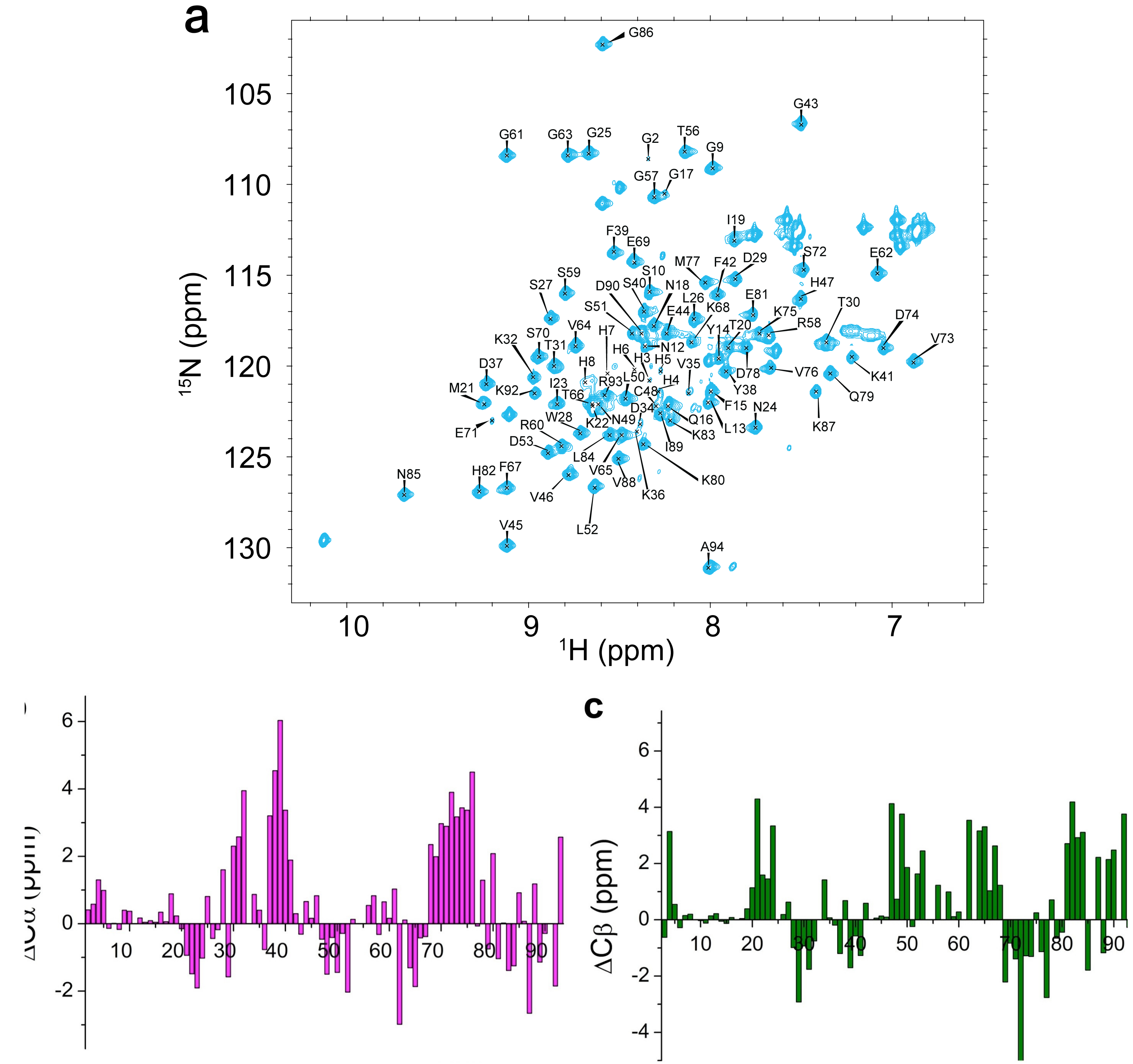
a
105
110
115
120
125
130
^{15}N (ppm)
10
9
8
7
^{1}H (ppm)
G86
G43
G61
G63
G25
G2
T56
G9
G57
G17
I19
F39
E69
M77
F42
D29
S72
E62
S59
S10
S27
D90
S40
N18
H47
S51
L26
K68
E81
T30
K75
D74
E44
Y14
T20
R58
V64
H7
S70
K32
T31
N12
V73
H6
H3
H5
D37
H8
V35
D78
V76
K41
L50
H4
R93
M21
K92
I23
Y38
T66
C48
Q79
F15
N24
K87
D34
W28
N49
Q16
E71
R60
K22
L13
K83
D53
I89
L84
K80
V65
N85
H82
F67
V88
K36
V46
L52
V45
A94
6
4
2
0
-2
10
20
30
40
50
60
70
80
90
c
ΔCβ (ppm)
-4

d

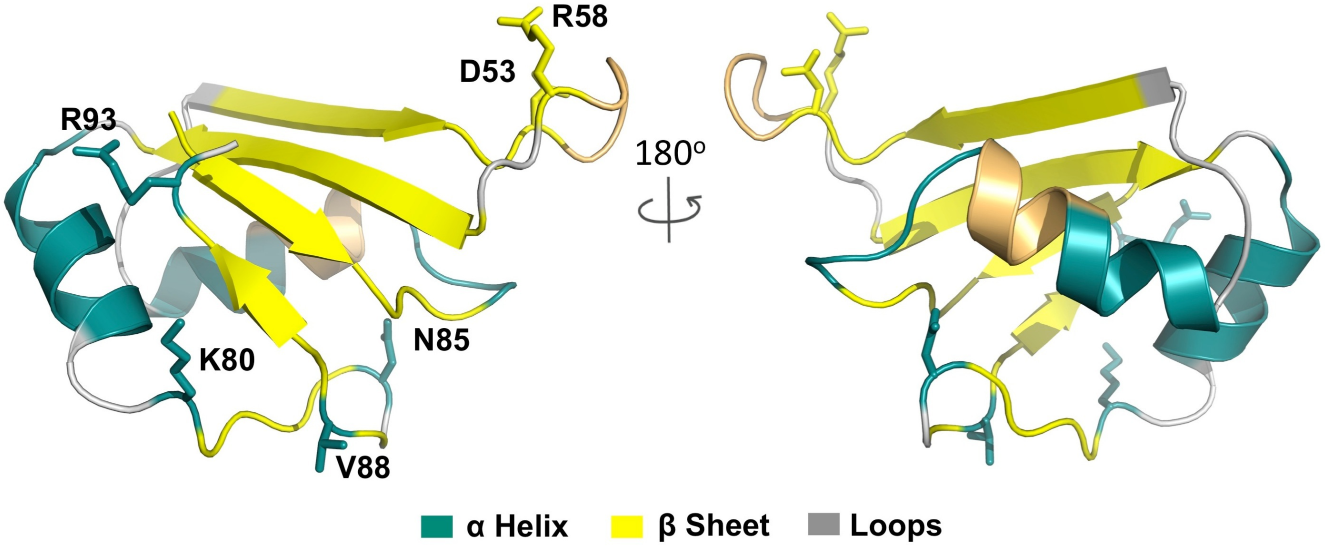

Supplementary Figure 9. NMR characterization of C1S1 conformation in solution. a, ^{1}H-^{15}N HSQC spectrum of C1S1. The assignments were determined on Sparky and MARS using HSQC, HNCACB, and CBCA(CO)NH spectra.; **b-c,** Differences of chemical shifts (ΔCα and ΔCβ) were calculated for each residue by subtracting the experiment chemical shifts of Cα and Cβ from the random coil values (see Methods and Fig. 2c). **d,** The residues that adopt α-helical (ΔCα-ΔCβ > 1 ppm) or β-sheet (ΔCα-ΔCβ < -1 ppm) structures are indicated on the C1S1 design model in dark cyan and yellow, respectively. Gray-colored regions indicate conformation of coils (|ΔCα-ΔCβ| < 1ppm) and they mostly reside in the loops. Because the His-tag was adjacent to grafted CONE 1 residues (unlike other designs), the NMR study was performed on His-tagged C1S1. The His-tag adopted random-coil conformation and did not interfere with the β-sheet conformation of CONE 1 residues.

In the loop connecting β13 and β12 (52-LDPITGR-60), D53 and R58 (shown as sticks) adopted a β-sheet conformation, probably due to formation of salt bridge. In the C terminal loop (79-QKEHKLNGKV-88), K80, N85, V88, and R93 (shown as sticks) adopted α-helical conformation (they form 'kinks' in the computational model). The wheat colored regions (33-KDVKD-37 and 52-LDPITGR-60) indicate assignments of medium or low confidence. T31, K33, P54, I55, and P91 could not be assigned. The conformations of these two segments also feature high plasticity in simulations (Supplementary Fig. 6). G17, G25, G43, G57, G61, G63, and G86 have no Cβ and their ΔCα-ΔCβ values were not calculated. Source data are provided as a Source Data file.

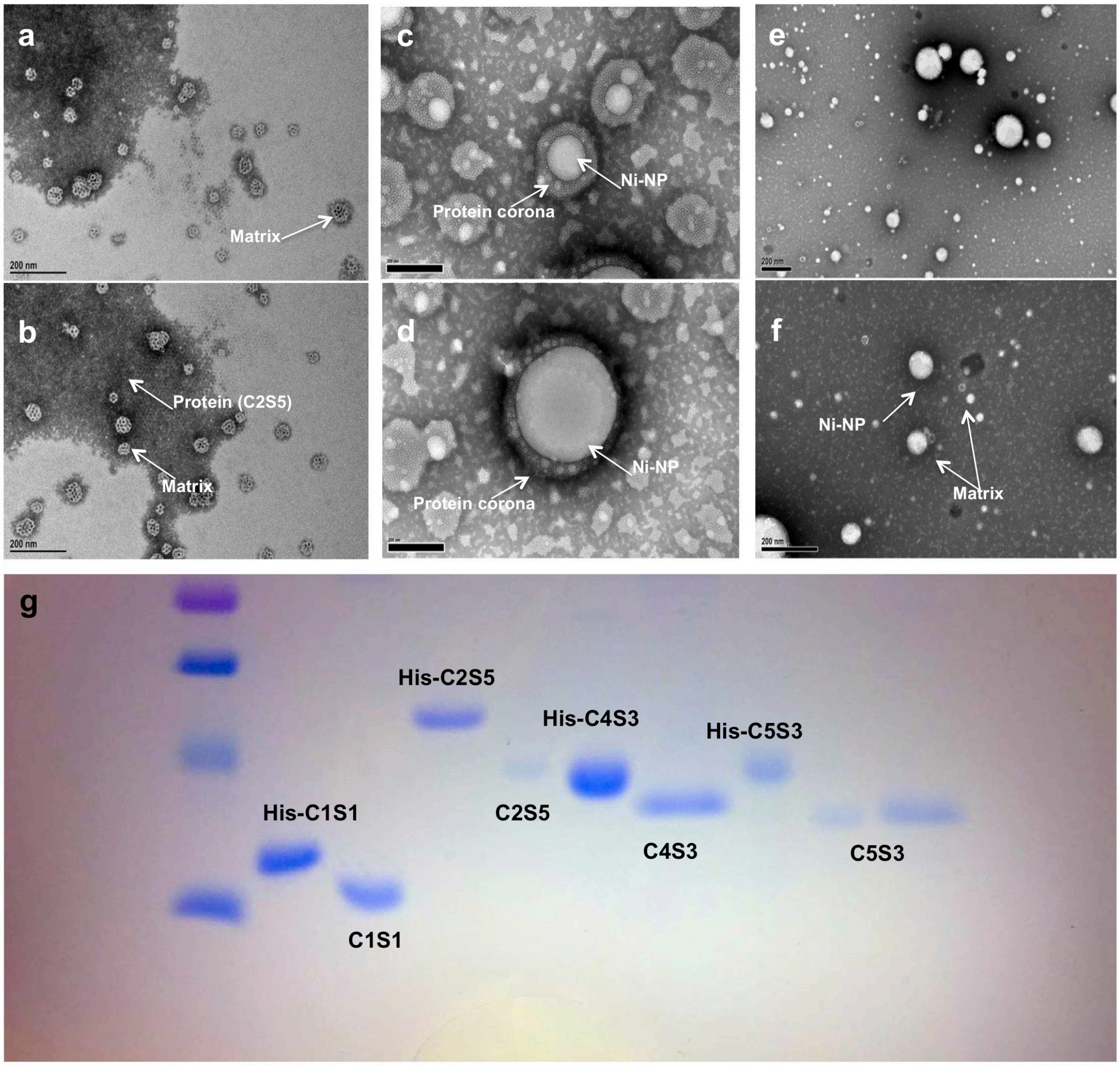

Supplementary Figure 10. Characterization of the immunogens used in rabbit immunization. a-f, Representative tunneling electron microscopy imaging of nanoparticles (NP): **a, b** Matrix-M adjuvant + C2S5 protein, **c, d** NPs + C2S5 protein, and **e, f** NPs + C2S5 protein + Matrix-M adjuvant. Scale bars in all panels, 200 nm. **g,** His-tagged immunogens were used with nanoparticles and His-tag-cleaved proteins were used with incomplete Freund`s adjuvant.

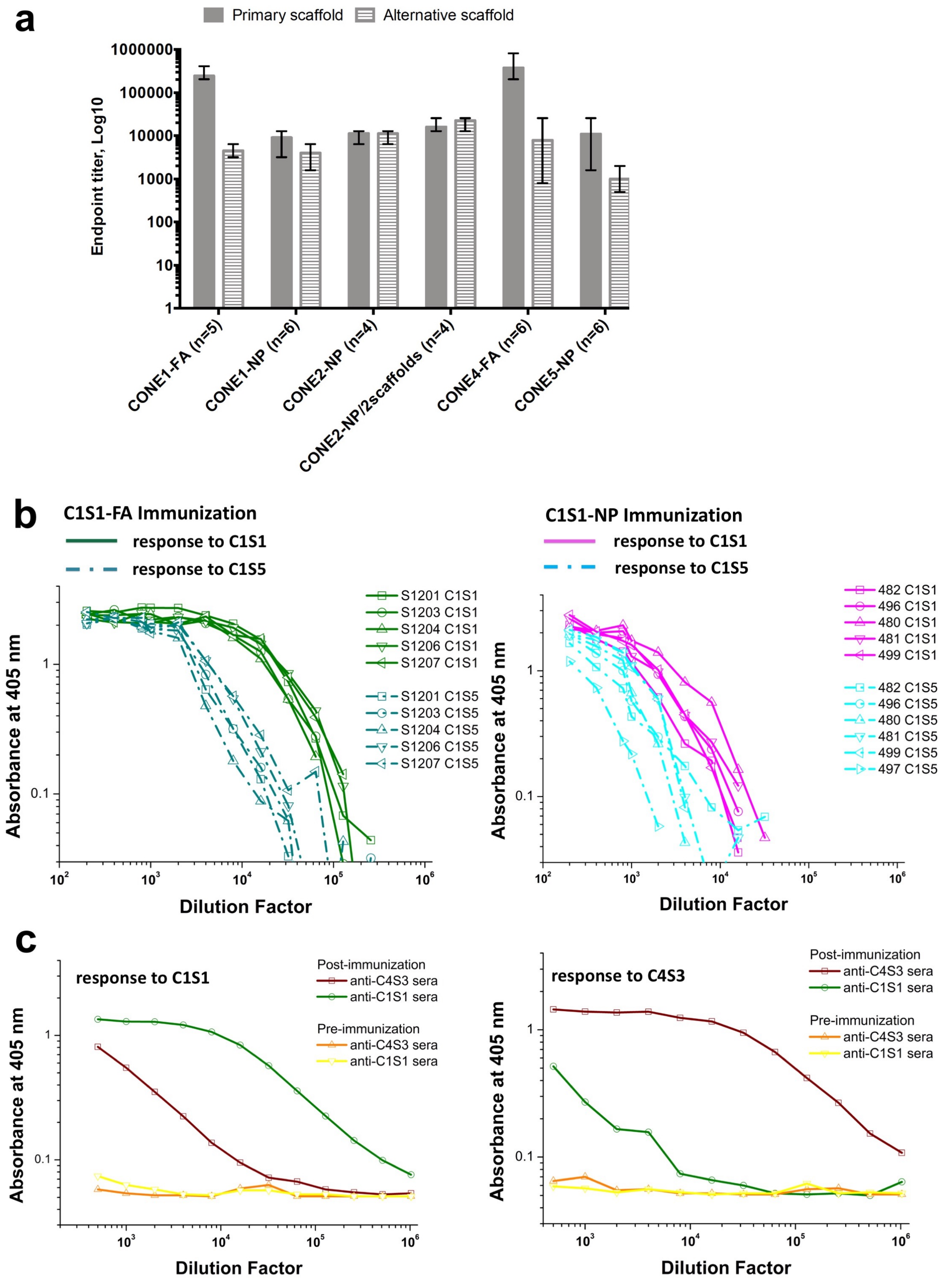
a
Primary scaffold
Alternative scaffold
Endpoint titer, Log10
1000000
100000
10000
1000
100
10
1
CONE1-FA (n=5)
CONE1-NP (n=6)
CONE2-NP (n=4)
CONE2-NP/2scaffolds (n=4)
CONE4-FA (n=6)
CONE5-NP (n=6)
b
C1S1-FA Immunization
response to C1S1
response to C1S5
S1201 C1S1
S1203 C1S1
S1204 C1S1
S1206 C1S1
S1207 C1S1
S1201 C1S5
S1203 C1S5
S1204 C1S5
S1206 C1S5
S1207 C1S5
Absorbance at 405 nm
Dilution Factor
C1S1-NP Immunization
response to C1S1
response to C1S5
482 C1S1
496 C1S1
480 C1S1
481 C1S1
499 C1S1
482 C1S5
496 C1S5
480 C1S5
481 C1S5
499 C1S5
497 C1S5
Absorbance at 405 nm
Dilution Factor
c
response to C1S1
Post-immunization
anti-C4S3 sera
anti-C1S1 sera
Pre-immunization
anti-C4S3 sera
anti-C1S1 sera
Absorbance at 405 nm
Dilution Factor
response to C4S3
Post-immunization
anti-C4S3 sera
anti-C1S1 sera
Pre-immunization
anti-C4S3 sera
anti-C1S1 sera
Absorbance at 405 nm
Dilution Factor

Supplementary Figure 11. ELISA characterization of the rabbit sera and interactions to CONE immunogens. a, Responses of sera to primary scaffolds (C1S1, C2S5, C4S3, and C5S3) and alternative scaffolds (C1S5, C2S3, C4S2, and C5S2). The average titer of a group of rabbit sera from the same CONE-immunization was reported. Two immunogens designed for the same CONE share the substructure formed by the epitope itself, while the rest of the protein is different (see Supplementary Figure 5). FA: incomplete Freund`s adjuvant; NP: nanoparticles. n=number of rabbits (4-6, indicated for each immunogen). Error bars represent standard deviation. **b,** Examples of ELISA characterization of anti-C1S1 sera. Each line represented the sera sample from a rabbit immunized with C1S1 (rabbit ID indicated as S1201, 481, *etc*.). The responses to C1S1 and the alternative scaffold (C1S5) were compared. Since C1S1 and C1S5 share the similarity only at the grafted epitope region, the responses of anti-C1S1 sera to C1S5 may indicate specificity to the CONE 1 epitope. **c,** The anti-C1S1 sera reacted weakly to C4S3, as did the anti-C4S3 sera to C1S1, suggesting their specificities to respective epitopes. The responses from pre-immunization sera were viewed as the background signal. Source data are provided as a Source Data file.

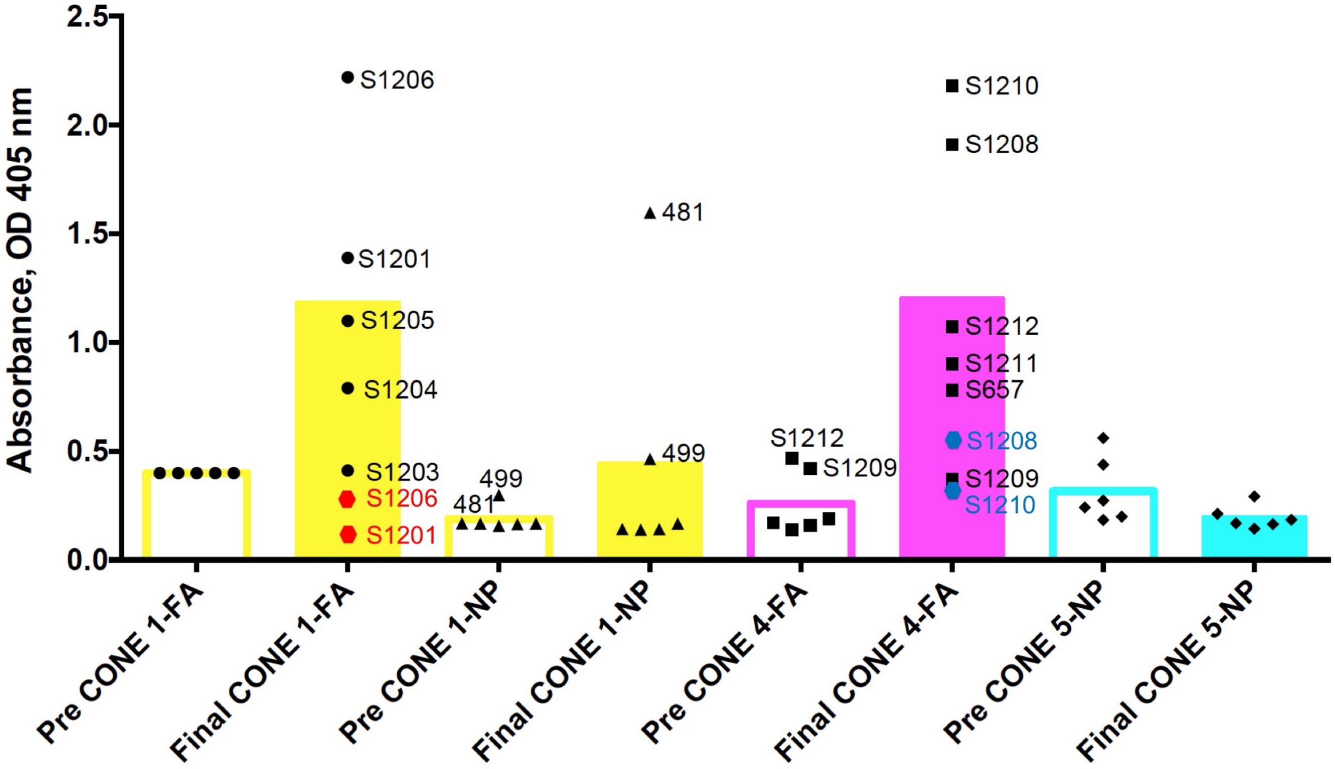

Supplementary Figure 12. Binding of immune sera to the deglycosylated Env protein (SOSIP) evaluated by ELISA. The sera were harvested in final immunization and tested at a 1:800 dilution. The signal from serum of individual rabbits were plotted (rabbits ID indicated as S1201, 481, *etc*.) The responses from the pre-immunization sera are shown as controls (response = 0.2 - 0.5). The sera S1206, S1201, S1210, S1208, *etc*. featured elevated binding capacity to Env (response = 1.0 - 2.3). For comparison, their responses to glycosylated SOSIP were shown as red and blue dots (1:800 dilution, response = 0.1 - 0.5). The glycan shield hindered the sera`s reactivity to Env, while the existence of glycan holes on the surface of deglycosylated Env rendered potential susceptibilities to the sera. Source data are provided as a Source Data file.

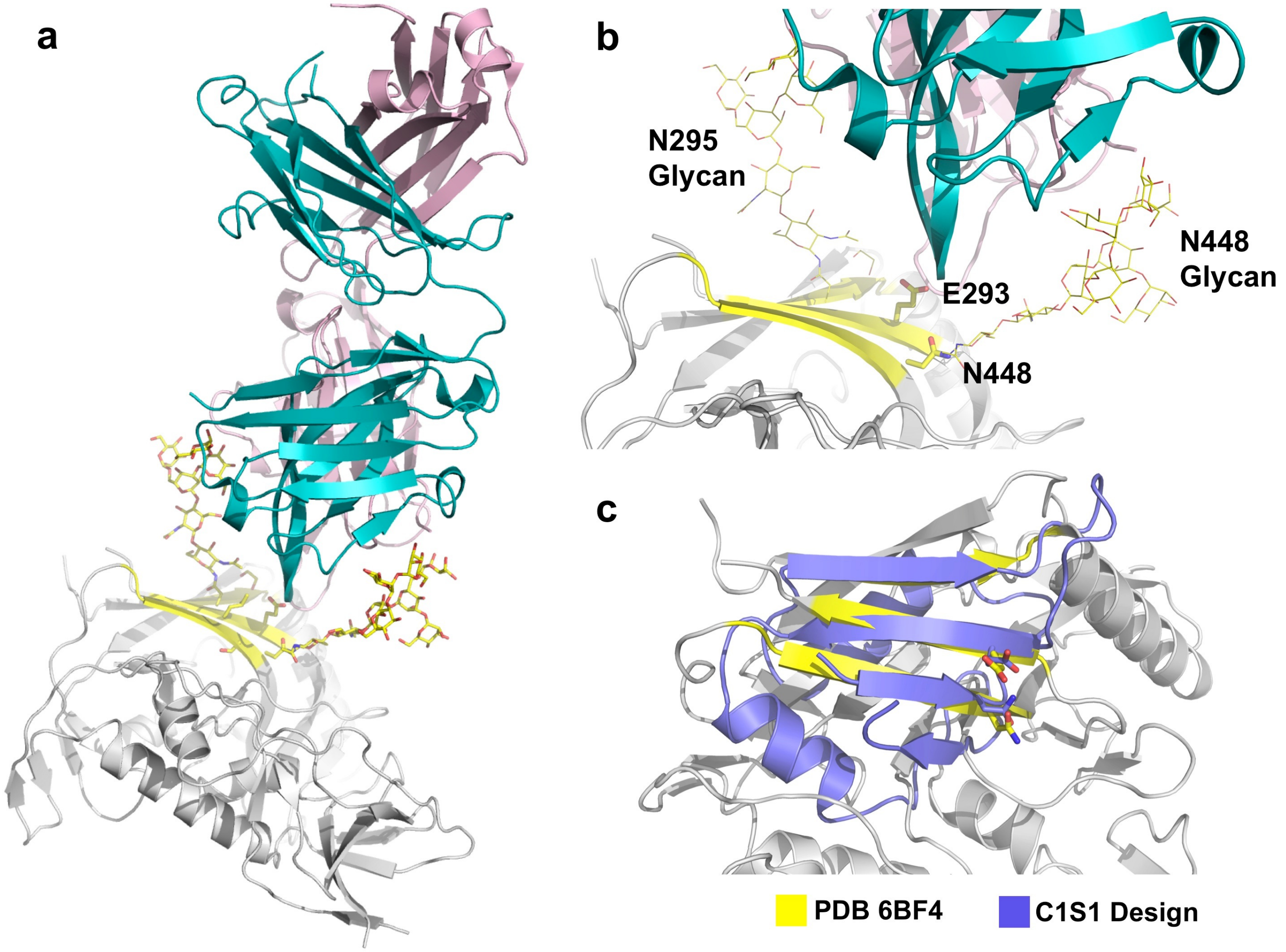

Supplementary Figure 13. The neutralizing antibody VRC-PG05 recognizes CONE 1-related epitopes. a, Crystal structure (PDB 6BF4) of VRC-PG05 Fab and gp120 core (truncated version of full-length gp120). The CONE 1-related regions were highlighted in yellow. **b,** Close-up view of VRC-PG05 epitopes, including E293, N448, and two glycans. **c,** Alignment between VRC-PG05 epitope and C1S1 design model. The conformation of grafted Glu and Asn residues (shown as sticks, slate) resembles the conformation of gp120 residues (yellow).

Supplementary Table 1. Scaffolds chosen in computational protein design.

CONE	ID	PDB	Methods	Protein Name
CONE 1<br>β 12/13/22	C1S1	1HD1	NMR	Ribonucleoprotein RNA-binding domain
	C1S2	2JYA	NMR	Uncharacterized protein ATU1810
	C1S3	2HLT	NMR	Probable acyl phosphatase
	C1S4	1E09	NMR	Cherry allergen Pru av 1
	C1S5	1ZO0	NMR	Antizyme isoform 1 from rat
CONE 2<br>α2 Helix	C2S1	2H60	NMR	Transcription activator SNF2L4 bromodomain
	C2S2	2FEK	NMR	Tyrosine phosphatase Wzb
	C2S3	2Z15	X-Ray	Human Tob1
	C2S4	1UNP	X-Ray	Protein kinase B pleckstrin homology domain
	C2S5	1BZ4	X-Ray	Apolipoprotein E
CONE 4<br>Loop C	C4S1	2FI9	X-Ray	Bartonella henselae outer domain protein
	C4S2	1N7E	X-Ray	AMPA receptor interacting protein GRIP 6th PDZ domain
	C4S3	2W4<br>C	X-Ray	Acylphosphatase 1
CONE 5<br>Loop E	C5S1	2CG<br>Q	X-Ray	Acy carrier protein ACPA
	C5S2	1P1L	X-Ray	Periplasmic cation tolerance protein CUTA
	C5S3	2CE7	X-Ray	Mitochondrial acyl carrier protein

Supplementary Table 2. Sequences of designed proteins‡.

Protein ID	
C1S1§	NITMKINGLSWDTTKKDVKDYFSKFGEVVHCNLSLDPITGRSRGEGVVTFKESESVDKVMDQKEHKLNGKVID<br>PKRA
C1S2	QGHMHANISRPAKTAMQSGTAKTNVWELVFTAEVPRKIDPIMGYTSSSDMNQTVKLNFETQEQAEAYAQRKG<br>IEYRVILPKEATRKVVSYTDNFRFNRTQPWTHGS
C1S3	NLTYKINVDGRVQGVGFRYFVQMEADKRKLAGNVSNRDDGRVEIVATGPENALQSFVEAVKNGSPFSKVTDI<br>SVTESRSLEGHHRFSIVY S
C1S4	GNFTYKSNFTSEIPPPRLFKAFVLDADNLVPKIAPQAIKHSEILEGDGGPGTIKKITFGEGSQYGYVKHKIDSIDKE<br>NYSYSYTLIEGDALGDTLEKIHYNTSLVASPSGGSIIESVSTYHTKGNVEIKEEHVKAGKEKASNLFKLIETYLK<br>GHPDAYN
C1S5	ILYSDERLHVNESPTSNDKTRELVITCTLTEAKNVTWKANWNGGGLYIELPAGPLPEGSKDSFAALLEFAEEQL<br>RADHVFICFPKNREDRAALLRTFSFLGFEIVRPGHPLVPKRPDACFMVYTLE
C2S1	MSPNPPNLTKKMKKIVDAVIKYKDSSSGRQLSEVFIQLPSRKELPEYYELIRKPVDFKKIKERIRNHKYRSLNDL<br>EKDVMLLCQNWQTFNLEGSLIYEDSTVLNKVFQRVREKIELVPRGSLEHHHHHH
C2S2	SHMFNNILVVCVGNICRSPTAERLLQRYHPELKVESAGLGALVGKGADPTAISVAAEHQLSLEGHCARQISRRL<br>CRNYDLILTMEKRHIERLCEMAPEMRGKVMLFGHWDNECEIPDPYRKSRETFTKVYKL<br>LQRSAEKWAQALNAEQV
C2S3	SSGMQLEIQVALNFIISYLYNKLPRRRVTKFNKELQRLLEKKYEGHWYPEKPYKGSGFRCIHIGEKVDPVIEQAS<br>KESGLDIDDVRGNLPQDLSVWIDPFEVSYQIGEKGPVKVLYVDDNSGPSSG
C2S4	MSDVAIVKEGWLHKRGEYIKTWRPRYFLLKNDGTFIGYKERPQDVDQREAPLNNFSVAQCQLMKTERPRPNT<br>FIIRCLQWTTVIERTFHVETPEERTEWNKAIQRVAEKLKKQEEEEMDFR
C2S5	SGQRWELALGRFWDYLRWVQTLSEQVQEELLSSQVETKLNKLMQRTMEELKAYKSELEEQLTPVAEETRARL<br>SKELQAAQARLGADMEDVCGRLVQYRGEVQAMLGQSTEELRVRLATHLNKLRQRLLEDADDLQKRLAVYQ<br>AG
C4S1	MSHAIQIREAHFPGRAPIDAYGNGGFRFADMSHRGSIICIPSGIYGIDWRGPVPTQEDISRVLEESDQIEVLLLGTE<br>EELLRLPEELRVLLWEKRISSDTMSTGAAVRTFNVLLAEDRAVAALLFAVE
C4S2	SSGAIIYTVELLREEEPLGITISGTEEPFDPIIISSLTKGGLAERTGAIHIGDRILAINSSSLKGKPLSEAIHLLQMAGG<br>VITLKIKKQTDAQPASS
C4S3	MAEGNTLISVDYEIFGKVQGVFFRKHTQAEGKKLGLVGWVQNTDRGTVQGQLQ<br>GPISKVRHMQEWLETRGSPKSHIDKANFNNEKLIEELDYSDFQIVA
C5S1	MKHHHHHHPMSDYDIPTTENLYFQGAMEEAINATIKEILPTNKTITANQVLVDTYGFDSLKLFQLITTLEDTFDI<br>AISFRDAQNI KTVGDVYTSVAVWFPETAKPAPLGKGTA
C5S2	MHNFIYITAPSLEEAERLAKRLLEKKLAACVNIFPIKSFFWWEGKIEAATEFAMIVKTRS<br>EKFAEVRDEVKEHHPYNKTCIDAIPIERGLKEFLDWIDETVE
C5S3	GANETTKQESPVVDTDINAVTNYIVGMCKEFLPKNKTVTPSSKLEELTQSEDRTWDCLDTVEFVLDVEEIF<br>DVTVPDEVADNFQTLQEIADFVVSERAKAGKFMKDQ

‡ The CONE-related epitopes were highlighted in yellow. A glycine remained as the N-terminus residue in TEV-cleaved protein samples. For proteins with Hi- tags, the sequence of MGHHHHHHGSENLYFQG was added to the N-terminal.

§ The proteins successfully purified and tested with biophysical assays were marked in red color. The alternative scaffolds of each CONE were also indicated with underlines.

Supplementary Table 3. Designed immunogens used in rabbit immunization.

Immunogen	Env Region	Dose	Adjuvant	No. of Rabbits
C1S1	β 12/13/22	300 μg	FA‡	5
C1S1	β 12/13/22	300 μg	NP	6
C2S5	α2 helix	300 μg§	NP	4
C2S5+C2S3	α2 helix	300 μg§	NP	4
C4S3	loop C	300 μg	FA	6
C5S3	loop E	300 μg	NP	6

‡ FA: incomplete Freund's adjuvant, NP: nanoparticle.

§ Immunization plan was amended after 8 weeks, and the amount of immunogen was increased from initial 30 μg (Matrix-M adjuvant) to 300 μg (nanoparticle).

Supplementary Table 4. Characterization of NPs used in rabbit immunization.

Ni-NP Conc.	Particle size (90º) (nm)‡	Ni-NPs (Particles /mL)	Zeta potential (mV)
1x (7.5 mg/mL)	109.7 ± 11.3	11.1 x 10^12	-15.5 ± 2.1
2x (15 mg/mL)	138.3 ± 11.6	17.4 x 10^12	-12.2 ± 1.3
5x (37.5 mg/mL)	152.0 ± 14.0	41.7 x 10^12	-13.5 ± 0.8

Ni-NP Conc.	C1S1 Conc. ($\mu g\ ml^{-1}$)	% Conjugation§
1x (7.5 mg/mL)	150	87.5%
	240	91.2%
	300	85.8%
	200	89.8%
2x (15 mg/mL)	240	93.3%

Ni-NP Conc.	C2S5 Conc. (μg mL^{-1})	% Conjugation
1x (7.5 mg/mL)	150	89.8%
	240	91.6%
	300	93.1%
	400	90.7%
	500	87.9%
2x (15 mg/mL)	300	96.2%

Ni-NP Conc.	C5S3 Conc. (μg ml^{-1})	% Conjugation
	240	90.3%
	300	89.2%
	400	76.5%
2x (15 mg/mL)	240	92.4%

‡ NanoSight analysis to quantify the number of NPs in a 7.5 mg mL^{-1} of solution of NPs.

§ Conjugation optimization of His-tagged proteins to NPs determined by UV analysis at 280 nm.

Supplementary Table 5. Immunogenicity of loop C linear peptide.

**Rabbit ID**‡	PA6606	PA6607	PA6608	PA6609	PA6610	C4S3
**ELISA End Point Titer**§	1 600	1 600	1 600	25 600	12 800	409 600

‡ Five rabbits were immunized with a synthetic peptide with the loop C sequence (CONE 4). The titers of rabbit sera were measured at day 56 and compared to that of C4S3-immunized rabbits at the same time point.

§ Sera titers are reported as the reciprocal of serum dilution, and values are calculated by measuring the dilution point where the absorbance drops below 0.2 at OD405 (4 times background).
