# Supplementary material for: Rationally designed carbohydrate-occluded epitopes elicit HIV-1 Env-specific antibodies
Source: Nat Commun. 2019 Feb 27;10:948. doi: 10.1038/s41467-019-08876-w (PMC6393580; doi:10.1038/s41467-019-08876-w)
Supplement: Supplementary file 2 — Reporting Summary [file 41467_2019_8876_MOESM2_ESM.pdf]

nature research

Corresponding author(s): Nikolay V. Dokholyan Ronald Swanstrom

Last updated by author(s): Jan 24, 2019

Reporting Summary

Nature Research wishes to improve the reproducibility of the work that we publish. This form provides structure for consistency and transparency in reporting. For further information on Nature Research policies, see Authors & Referees and the Editorial Policy Checklist.

Statistics

For all statistical analyses, confirm that the following items are present in the figure legend, table legend, main text, or Methods section.

n/a	Confirmed	
☐	☒	The exact sample size (*n*) for each experimental group/condition, given as a discrete number and unit of measurement
☐	☒	A statement on whether measurements were taken from distinct samples or whether the same sample was measured repeatedly
☒	☐	The statistical test(s) used AND whether they are one- or two-sided<br>*Only common tests should be described solely by name; describe more complex techniques in the Methods section.*
☒	☐	A description of all covariates tested
☒	☐	A description of any assumptions or corrections, such as tests of normality and adjustment for multiple comparisons
☒	☐	A full description of the statistical parameters including central tendency (e.g. means) or other basic estimates (e.g. regression coefficient) AND variation (e.g. standard deviation) or associated estimates of uncertainty (e.g. confidence intervals)
☒	☐	For null hypothesis testing, the test statistic (e.g. *F*, *t*, *r*) with confidence intervals, effect sizes, degrees of freedom and *P* value noted<br>*Give P values as exact values whenever suitable.*
☒	☐	For Bayesian analysis, information on the choice of priors and Markov chain Monte Carlo settings
☒	☐	For hierarchical and complex designs, identification of the appropriate level for tests and full reporting of outcomes
☒	☐	Estimates of effect sizes (e.g. Cohen's *d*, Pearson's *r*), indicating how they were calculated

Our web collection on statistics for biologists contains articles on many of the points above.

Software and code

Policy information about availability of computer code

Data collection	HKL 2000 (for crystallography), Gromacs (for MD simulation), Erebus/Eris/Chiron web servers (for protein design); see Method section for detail.
Data analysis	PHENIX, CCP4, and Molprobity (for crystallography); Sparky, MARS (for NMR); Prism 6 (for ELISA and neutralization); see Method section for detail.

For manuscripts utilizing custom algorithms or software that are central to the research but not yet described in published literature, software must be made available to editors/reviewers. We strongly encourage code deposition in a community repository (e.g. GitHub). See the Nature Research guidelines for submitting code & software for further information.

Data

Policy information about availability of data

All manuscripts must include a data availability statement. This statement should provide the following information, where applicable:

- Accession codes, unique identifiers, or web links for publicly available datasets
- A list of figures that have associated raw data
- A description of any restrictions on data availability

The source data underlying Figs. 2c, 3, 4 and Supplementary Figs. 6, 7, 8, 9, 11 and 12 are provided as a Supplementary Source Data file. The coordinates of the designs C2S5 and C4S3 are available from the RCSB Protein Data Bank with the accession codes 6CFE and 6CBU[. A reporting summary for this Article is available as a Supplementary Information file. All other data supporting the findings of this manuscript are available from the corresponding authors (N.V.D. and R.S.) upon reasonable request.

Field-specific reporting

Please select the one below that is the best fit for your research. If you are not sure, read the appropriate sections before making your selection.

☒ Life sciences ☐ Behavioural & social sciences ☐ Ecological, evolutionary & environmental sciences

For a reference copy of the document with all sections, see nature.com/documents/nr-reporting-summary-flat.pdf

Life sciences study design

All studies must disclose on these points even when the disclosure is negative.

Sample size	small groups of rabbits (four to six per group) were immunizated with the designed immunogens to raise antibodies.
Data exclusions	n/a
Replication	Antibody titers were determined by ELISA during the immunization process. The data represent the mean titers for each group of rabbits. n (4-6) demonstrating the number of rabbits in the immunization. For neutralization assays, each experiment (different combinations of rabbits, sera, and pseudotyped HIV-1 mutants) was repeated three times.
Randomization	n/a
Blinding	n/a

Reporting for specific materials, systems and methods

We require information from authors about some types of materials, experimental systems and methods used in many studies. Here, indicate whether each material, system or method listed is relevant to your study. If you are not sure if a list item applies to your research, read the appropriate section before selecting a response.

Materials & experimental systems

n/a	Involved in the study	
☐	☒	Antibodies
☐	☒	Eukaryotic cell lines
☒	☐	Palaeontology
☐	☒	Animals and other organisms
☒	☐	Human research participants
☒	☐	Clinical data

Methods

n/a	Involved in the study	
☒	☐	ChIP-seq
☒	☐	Flow cytometry
☒	☐	MRI-based neuroimaging

Antibodies

Antibodies used	Total IgG fraction purified from rabbit sera
Validation	SDS-PAGE and Western blotting

Eukaryotic cell lines

Policy information about cell lines

Cell line source(s)	HEK293T and HEK293F were acquired from American Tissue Culture Collection; TZM-bl cells were obtained from NIH AIDS Reagent program.
Authentication	n/a
Mycoplasma contamination	n/a
Commonly misidentified lines (See ICLAC register)	n/a

Animals and other organisms

Policy information about studies involving animals; ARRIVE guidelines recommended for reporting animal research

Laboratory animals	New Zealand white rabbits were housed in accordance with the NIH Guide for the Care and Use of Laboratory Animals and the protocol approved by the Institutional Animal Care and Use Committee of UNC-Chapel Hill.
Wild animals	n/a
Field-collected samples	n/a
Ethics oversight	UNC Chapel Hill Institutional Animal Care and Use Committee

Note that full information on the approval of the study protocol must also be provided in the manuscript.
